# Structural connectome quantifies tumour invasion and predicts survival in glioblastoma patients

**DOI:** 10.1093/brain/awac360

**Published:** 2022-10-03

**Authors:** Yiran Wei, Chao Li, Zaixu Cui, Roxanne Claudeve Mayrand, Jingjing Zou, Adrianna Leanne Kok Chi Wong, Rohitashwa Sinha, Tomasz Matys, Carola-Bibiane Schönlieb, Stephen John Price

**Affiliations:** Cambridge Brain Tumour Imaging Laboratory, Division of Neurosurgery, Department of Clinical Neurosciences, University of Cambridge, Cambridge CB2 0QQ, UK; Cambridge Brain Tumour Imaging Laboratory, Division of Neurosurgery, Department of Clinical Neurosciences, University of Cambridge, Cambridge CB2 0QQ, UK; Department of Applied Mathematics and Theoretical Physics, The Centre for Mathematical Imaging in Healthcare, Cambridge CB3 0WA, UK; Chinese Institute for Brain Research, Beijing 102206, China; Cambridge Brain Tumour Imaging Laboratory, Division of Neurosurgery, Department of Clinical Neurosciences, University of Cambridge, Cambridge CB2 0QQ, UK; Division of Biostatistics and Bioinformatics, Department of Family Medicine and Public Health, University of California, San Diego, CA 92103, USA; Cambridge Brain Tumour Imaging Laboratory, Division of Neurosurgery, Department of Clinical Neurosciences, University of Cambridge, Cambridge CB2 0QQ, UK; Cambridge Brain Tumour Imaging Laboratory, Division of Neurosurgery, Department of Clinical Neurosciences, University of Cambridge, Cambridge CB2 0QQ, UK; Department of Radiology, University of Cambridge, Cambridge CB2 0QQ, UK; Department of Applied Mathematics and Theoretical Physics, The Centre for Mathematical Imaging in Healthcare, Cambridge CB3 0WA, UK; Cambridge Brain Tumour Imaging Laboratory, Division of Neurosurgery, Department of Clinical Neurosciences, University of Cambridge, Cambridge CB2 0QQ, UK

**Keywords:** brain connectome, glioblastoma, tumour invasion, brain reorganization, survival analysis

## Abstract

Glioblastoma is characterized by diffuse infiltration into the surrounding tissue along white matter tracts. Identifying the invisible tumour invasion beyond focal lesion promises more effective treatment, which remains a significant challenge. It is increasingly accepted that glioblastoma could widely affect brain structure and function, and further lead to reorganization of neural connectivity. Quantifying neural connectivity in glioblastoma may provide a valuable tool for identifying tumour invasion.

Here we propose an approach to systematically identify tumour invasion by quantifying the structural connectome in glioblastoma patients. We first recruit two independent prospective glioblastoma cohorts: the discovery cohort with 117 patients and validation cohort with 42 patients. Next, we use diffusion MRI of healthy subjects to construct tractography templates indicating white matter connection pathways between brain regions. Next, we construct fractional anisotropy skeletons from diffusion MRI using an improved voxel projection approach based on the tract-based spatial statistics, where the strengths of white matter connection and brain regions are estimated. To quantify the disrupted connectome, we calculate the deviation of the connectome strengths of patients from that of the age-matched healthy controls. We then categorize the disruption into regional disruptions on the basis of the relative location of connectome to focal lesions. We also characterize the topological properties of the patient connectome based on the graph theory. Finally, we investigate the clinical, cognitive and prognostic significance of connectome metrics using Pearson correlation test, mediation test and survival models.

Our results show that the connectome disruptions in glioblastoma patients are widespread in the normal-appearing brain beyond focal lesions, associated with lower preoperative performance (*P* < 0.001), impaired cognitive function (*P* < 0.001) and worse survival (overall survival: hazard ratio = 1.46, *P* = 0.049; progression-free survival: hazard ratio = 1.49, *P* = 0.019). Additionally, these distant disruptions mediate the effect on topological alterations of the connectome (mediation effect: clustering coefficient −0.017, *P* < 0.001, characteristic path length 0.17, *P* = 0.008). Further, the preserved connectome in the normal-appearing brain demonstrates evidence of connectivity reorganization, where the increased neural connectivity is associated with better overall survival (log-rank *P* = 0.005).

In conclusion, our connectome approach could reveal and quantify the glioblastoma invasion distant from the focal lesion and invisible on the conventional MRI. The structural disruptions in the normal-appearing brain were associated with the topological alteration of the brain and could indicate treatment target. Our approach promises to aid more accurate patient stratification and more precise treatment planning.

## Teaser

Wei *et al.* characterize topological changes in the connectome in patients with glioblastoma. Distant connectome disruptions—invisible on conventional MRI—are widespread beyond the focal lesions and are associated with impaired cognition and survival. Such changes could have prognostic value in patients with glioblastoma.

## Introduction

Glioblastoma is the most common primary malignant brain tumour in adults, characterized by diffuse infiltration into the surrounding tissue.^1^ It is increasingly accepted that glioblastoma widely influences the brain structure and function beyond the focal lesion.^2,3,4,5^ Evidence shows that glioblastoma can induce profound reorganization of neural connectivity,^5,6^ while neuronal activity can promote tumour progression.^7^ This bidirectional interaction underscores the promise of characterizing neural connectivity for better understanding glioblastoma invasion, which may facilitate more accurate patient stratification for personalized management.

Diffusion-weighted MRI (dMRI) is a method to estimate the structural connectivity of the brain. It is more sensitive to detecting occult tumour invasion than conventional T_1_-weighted and fluid-attenuated inversion recovery (FLAIR) images.^8^ Evidence shows that dMRI can indicate the tissue signature of glioma,^9^ offering value to evaluate invasiveness,^10,11^ detect peritumoral invasion,^12,13^ indicate subventricular zone involvement^14^ and predict molecular phenotypes.^15^ These studies, however, have focused on the focal tumour, instead of the systematic disturbance of the brain.

The advance in neuroimaging has represented structural connectivity as a complex network, namely structural connectome.^16,17^ This approach models brain regions as nodes while the white matter (WM) connections among brain regions as edges. Graph theoretical analysis of the derived structural networks shows to characterize various neurological and psychiatric disorders.^18,19,20^ Moreover, studies suggest that brain tumours may alter the connectome topology while brain networks correspondingly demonstrate robustness and reorganization.^5–21,22,23^ Recent evidence shows that the topological features derived from the connectome may predict tumour location frequency in glioma patients^24^ and appear to predict patient survival.^25^ However, it remains largely unknown whether the connectome disruption could be quantified for patient stratification. Of particular significance is whether the robustness of the connectome could affect patient outcome.

The purpose of this study was to characterize the disruption of the structural connectome in glioblastoma patients and investigate the clinical significance. We hypothesized that glioblastoma could induce both focal and global disturbance to the structural connectome, leading to topological alteration of the brain and impacting patient outcomes. We tested this hypothesis in two prospective glioblastoma cohorts. First, we constructed the structural networks using the dMRI from glioblastoma patients and healthy controls. Second, we quantified the focal and distant disrupted connectome separately from each patient. Third, we calculated the disruption indices and topological features and examined their significance in survival models. Last, we modelled the alteration of the preserved connectivity after removing the disrupted connectome and investigated its significance on patient survival. The results revealed widespread disruptions of the structural connectome, which could lead to topological alterations and show prognostic value in glioblastoma patients.

## Materials and methods

### Subjects

Patients with a radiological diagnosis of *de novo* supratentorial glioblastoma were prospectively recruited for resection (discovery: July 2010–August 2015; validation: July 2017–October 2019) by the multidisciplinary team central review. For both cohorts, patients were consecutively recruited following identical inclusion and exclusion criteria, with data prospectively collected (see Supplementary methods). All patients underwent preoperative 3D magnetization-prepared rapid acquisition gradient echo (MPRAGE) [pre-contrast (T_1_) and post-contrast (T_1C_)], T_2_-weighted FLAIR and dMRI sequences. Patient preoperative cognitive performance was tested using the Mini-Mental State Examination (MMSE), dichotomized as <27 or ≥27 as reported.^26^

We included a control cohort with dMRI and T_1_ sequences available from https://brain-development.org/ixi-dataset/. We also included the high angular resolution dMRI from the Alzheimer’s Disease Neuroimaging Initiative (ADNI, http://adni.loni.usc.edu/) for constructing an unbiased template of WM connection with high spatial resolution.

### Treatment

All patients underwent maximal safe surgery using 5-aminolevulinic acid fluorescence (5-ALA, Medac) and neuro-navigation (StealthStation, Medtronic). For maximal safe resection in both study cohorts, where appropriate, other adjuvants, including awake surgery, cortical and subcortical mapping and intraoperative electrophysiology were also applied. According to the postoperative MRI within 72 h, the extent of resection was assessed as complete or partial resection of enhancing tumour or biopsy. Adjuvant therapy was determined by the multidisciplinary team as standard based on patient postoperative status. All patients were followed up according to the response assessment in neuro-oncology criteria. Overall survival (OS) and progression-free survival (PFS) were used as endpoints.

### Tumour segmentation

All anatomical MRI (T_1_, T_2_ and FLAIR) were co-registered to T_1C_ images with an affine transformation, using the linear image registration tool (FLIRT) function in the FMRIB Software Library (FSL).^27^ To segment the tumour, we applied a multi-scale 3D Deep Convolutional Neural Network,^28^ implemented in the Cancer Imaging Phenomics Toolkit (CaPTk, https://cbica.github.io/CaPTk/index.html). The manual correction was performed using 3D slicer v.4.6.2 (https://www.slicer.org/) by a neurosurgeon (C.L.) and a researcher (Y.W.) after an initial training period and reviewed by an experienced neuroradiologist (T.M.). The final consensus was achieved to ensure inter-rater reliability.

### Quantifying the brain connectome

**Figure 1:**
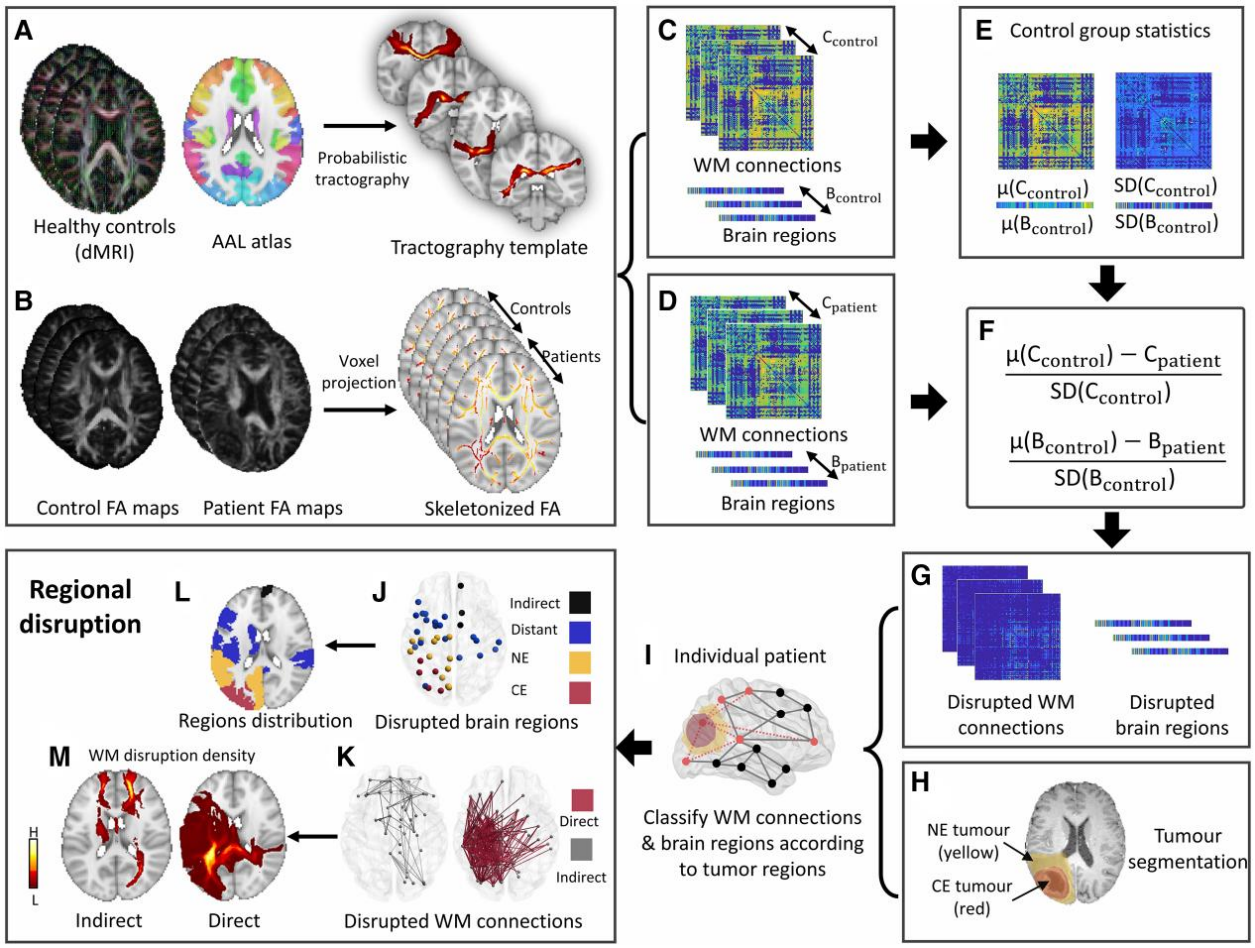
**Study design.** (**A**) Probabilistic tractography is performed on the high-resolution dMRI of 10 healthy controls to generate a template for the WM connections among the 90 regions on the Automatic Anatomical Labelling atlas. (**B**) Skeletonized fractional anisotropy (FA) maps are generated from both patients and age-matched healthy controls to estimate the WM connection strength, using an improved voxel projection procedure based on TBSS. (**C** and **D**) The strengths of WM connections are derived in healthy controls and patients by combining WM connection template and skeletonized FA. The strengths of brain regions are calculated by aggregating the strengths of linking WM connections. (**E**–**G**) By comparing patients to controls, the disrupted connectome in patients is identified as the WM connection (C_patient_) or brain region (B_patient_) with a strength of over 2SD (95% confidence interval) lower than the mean strength of the control group [μ(C_control_) or μ(B_control_)]. The disruption indices of WM connections and brain regions are calculated by averaging the disruption matrices/vectors. (**H**) The lesion is segmented as contrast-enhancing (CE, red) and non-enhancing (NE, yellow) tumours. (**I**–**K**) The brain region disruptions are classified into CE, NE, distant and indirect disruptions. The WM connection disruption is classified into direct and indirect disruptions. (**L**) Categorical disrupted brain regions are mapped to the standard space. (**M**) Disrupted WM connections are mapped to the standard space producing a disruption density map.

Tractography is a technique to measure the strength of WM connection by tracking the fibre pathway connecting brain regions. However, directly performing tractography on the brain with tumours may cause tracking failure or artefacts, e.g. an unrealistic fibre belt surrounding the tumour.^29^ To bypass the bias of performing tractography on the lesioned brain, we used an approach to generate a template from healthy controls for tract localization and leveraged the voxel project step of tract-based spatial statistics (TBSS)^30^ to robustly estimate the strength of WM connection in patients by comparing patients to healthy controls.^31,32^ The complete pipeline includes the following steps: (i) constructing a group-based tract template (Fig. 1A); (ii) producing individualized skeletonized fractional anisotropy (FA) maps (Fig. 1B); (iii) combining the template with the FA skeleton maps to estimate the connectome strength in both patients and controls (Fig. 1C and D); and (iv) identifying the significantly disrupted connectome in patients (Fig. 1E–G).

#### Constructing a template of white matter connections

An unbiased WM connection template was generated by performing probabilistic tractography on the dMRI of the 10 healthy controls. In detail, the cortical/subcortical regions on dMRI were parcellated into 90 brain regions, using the Automatic Anatomical Labelling (AAL) atlas^33^ in the standard space.^34^ The AAL atlas included the grey-WM boundary to facilitate tractography. Deformable registration was performed using the Advanced Normalization Tools (ANTs).^35^

Eddy currents and subject motions in dMRI were corrected using the FSL eddy tool (v.6.0.0). A crossing fibre model was fitted to each control’s dMRI using the FSL function bedpostx. Probabilistic tractography between each pair of the 90 regions was subsequently performed using FSL Probtrackx2.^36^ Each region of interest (ROI) was used as a seed (starting ROI) and target (ending ROI) once in the tracking. For each pair of seed/target ROI, 5000 streamlines were sampled from the seed mask. Only the streamlines reaching the target mask were retained. The threshold of tracking curvature was set to 0.2 (80°). Streamline samples were terminated when they have travelled 2000 steps with step length of 0.5 mm or entered the cortical/subcortical regions. Streamlines were discarded if they entered the cerebrospinal fluids in the ventricle or re-entered the seed region.

Distribution maps were generated for all possible WM connections between the 90 cortical/subcortical regions in the healthy controls. The yielded distribution maps were non-linearly transformed to the MNI-152 space and averaged to mean connection distribution across the controls using fslmaths, which was thresholded to only retain the voxels with top 5% probability and finally binarized, providing conservative pathways for the template.

#### Generating skeletonized FA maps

In generating skeletonized FA maps, we included age-matched controls to reduce potential bias from ageing-related WM pathology. We fitted all dMRIs with a tensor model using the FSL diffusion toolbox.^37^ The yielded FA maps were non-linearly co-registered to a FA template in the standard space using the deformable function of ANTs, which could mitigate the brain deformation caused by tumour.^38^ This approach could outperform FNIRT^39^ (default registration tool in TBSS) in co-registering FA^40^ and pathology-bearing T_1_.^41^ To minimize potential the signal-noise ratio effect from different MRI protocols, we normalized the FA maps using the histogram-matching method.^42^

The local maxima voxels from the FA map of patients and controls were projected to this skeleton mask using an improved iterative TBSS projection approach guided by tract orientation.^43^ A FA skeleton mask in the standard space (FMRIB58 FA skeleton 1 mm) was used as the target for FA voxel projection.^44^ The generated individualized FA skeletons represent the centre integrity of WM tracts in subjects.

#### Estimating strengths of WM connections and brain regions

For each patient/control, the WM connection strength was calculated as the mean strength of all tract segments in the FA skeleton, constrained by the WM connection template. The columns and rows of each individualized connection matrix represent the brain regions, while the elements in the matrices (*C_ij_*) represent the strength of WM connection between the brain regions *i* and *j*. We calculated the strength *B*_*i*_ for region *i*, by aggregating the connectivity strength of the WM connection *C*_*ij*_ connected to this region.

#### Identification of significantly disrupted connectome

We first calculated the mean strength and standard deviation (SD) of each WM connection across all healthy controls, which was compared to individual patients (Fig. 1G). We defined the significantly decreased connectome in patients as those with a strength of over 2SD (95% confidence) lower than the mean strength of the whole control group.

### Estimating connectome disruptions

We calculated the patient-wise global disruption index by averaging the disruption of WM connections and brain regions, respectively. To address intra-tumour heterogeneity, we segmented the lesion as contrast-enhancing (CE, the entire area within the T_1_ CE rim) and peritumoral non-enhancing (NE, the hyper-intensities surrounding CE on FLAIR) subregions (Fig. 1H and I). Based on the segmented tumour, we categorized the disrupted WM connections and brain regions as follows (Fig. 1J and K):

#### Disrupted WM connections

- Direct disruption: directly disrupted by tumour, travelling across the contrasting enhancing or NE tumour.
- Indirect disruption: disrupted without crossing the lesion.

#### Disrupted brain regions

- Tumour disrupted regions: disrupted regions within the lesion, further categorized into CE or NE disrupted regions, according to the overlap with tumour subregions.
- Distant disrupted regions: disrupted regions within the normal-appearing brain and connected to the lesion via WM connections.
- Indirect disrupted regions: disrupted regions without any connection to the lesion.

Finally, we calculated the regional disruption index as the average of each category.

### Group distribution of tumour disruption

To generate a tumour frequency map, we non-linearly transformed all tumour masks from individual patients to the standard space using ANTs, with voxel-wise tumour distribution density normalized at the group level. Regional disrupted WM connections and brain regions are mapped to the standard space for visualization (Fig. 1L and M). We also quantified the disruption probability of brain regions and major tracts among patients. For tracts, we mapped the WM connections to the 42 anatomical tracts constructed from the 1000 healthy subjects available in the XTRACT toolbox of FSL. The disruption probability for each brain region or tract was calculated as the percentage of patients with this brain region or tract disrupted. We also calculated the proportion of disrupted connectomes out of all the patients.

### Topological features of the connectome

We calculated two most commonly used topological features for each patient using the Brain Connectivity Toolbox^17^: characteristic path length and clustering coefficient. Briefly, the clustering coefficient measures the probability of two direct topological neighbours of a specific brain region being connected. The characteristic path length measures the average shortest path length of the network (see Supplementary material for formulas).^45^ We filtered the connectome with a population-consistency-based strength threshold to reduce the noise in feature calculation.^46^

### Preserved connectivity of disrupted distant regions

By removing the disrupted WM connections, we investigated the preserved connections, defined as the remaining WM connections to the disrupted distant regions. Through pairwise comparison between patients and age-matched controls, we categorized patients into two subgroups with overall increased or decreased connectivity respectively, according to the summed strength of the preserved connectivities.

### Statistical analysis

All analyses were performed in RStudio v.3.2.3 and MATLAB 2019b. The Benjamini–Hochberg procedure (BH–FDR) correction was performed for all multiple comparison tests. The hypothesis of no effect was rejected at a two-sided level of 0.05. The difference between global/regional disruptions and topological features of patient cohorts/subgroups were compared using two sample *t*-test. Pearson correlation was used to test the correlations between disruption indices, MMSE score, tumour volume and topological features. A multiple regression was used to identify significant regional disruptions contributing to topological changes. The mediation model was used to test whether the previously identified contributor mediated the effect of lesion on brain topology.

We performed survival analysis to evaluate disruption indices and topological features using OS and PFS. Patients alive at the last known follow-up were censored. Kaplan–Meier survival curves were compared using log-rank test, based on disruption indices and topological features dichotomized by either median or the optimal cut-off defined using the maximally selected rank statistics in R package ‘Survminer’,^47^ whichever was more significant.

In addition to the univariate Cox proportional hazards model, we conducted multivariate Cox proportional hazards model accounting for all relevant clinical covariates, including *O*-6-methylguanine-DNA methyltransferase (MGMT) methylation status, isocitrate dehydrogenase-1 (IDH-1) mutation, sex, age, the extent of resection, adjuvant therapy and tumour volume. We also included two tumour location features from the Visually Accessible Rembrandt Images (VASARI) feature set (https://wiki.cancerimagingarchive.net/display/Public/TCGA-GBM) describing the involvement of eloquent cortex and deep WM to account for the effects of tumour cortical/subcortical brain regions.

We used the receiver operating characteristic curves to evaluate the accuracy in predicting OS. We fit a generalized linear model and calculated the area under the receiver operating characteristic curve (AUC). The model included the significant disruption indices and topological features from the univariate Cox proportional hazards models.

### Data availability

Enquiries or requests for the data should be directed to the corresponding author.

## Results

### Subject characteristics

For the discovery cohort, we recruited 136 patients for preoperative MRI scanning. After excluding 19 patients according to the trial exclusion criteria, we finally included 117 of 136 (86.0%) patients (mean age 59 years, range 22–75 years, 89 males) for analysis. Six patients (5.1%) were lost to follow-up. The median OS was 392 (range 34–1932) days. The median PFS was 275 (range 13–1393) days.

For the validation cohort, 49 patients were initially recruited and 42 patients were finally included according to the trial exclusion criteria (mean age 59 years, range 28–75 years, 30 males). Ten patients (23.8%) were alive or lost to follow-up. The median OS was 335 (range 55–962) days. The median PFS was 246 (range 21–805) days. Two study cohorts showed no significant differences in clinical variables (Supplementary Table 1).

We included 117 healthy age-matched subjects (mean age 59.9 years, 78 males) from the IXI datasets as healthy controls. In addition, we included 10 healthy subjects (mean age 60.9 years, 5 males) from the ADNI for the tractography template. All cohorts showed no significant difference in age.

### Global disrupted connectome demonstrates clinical significance

**Figure 2:**
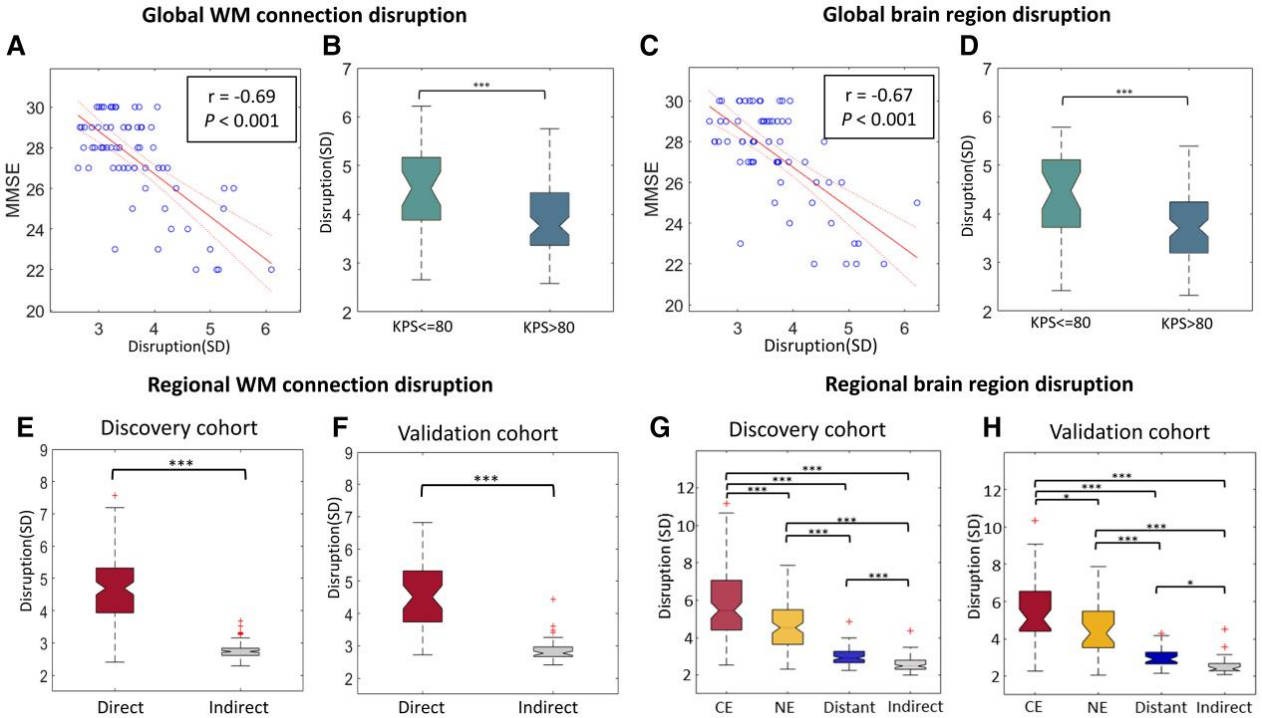
**The clinical significance of disruption indices.** (**A** and **C**) The global disruption indices of WM connections and brain regions are both negatively correlated with the MMSE score. (**B** and **D**) Higher disruption is associated with worse Karnofsky Performance Status. (**E** and **F**) In both discovery and validation cohorts, direct connection disruption is higher than Indirect disruption. (**G** and **H**) Disruption of tumour regions (CE, NE) are more significantly disrupted than the normal-appearing brain (distant, indirect). ***P < 0.001.

We observed that the global disruption of WM connections (Pearson: *r* = −0.67, *P* < 0.001; Spearman: rho = −0.48, *P* < 0.001; Fig. 2A) and brain regions (Pearson: *r* = −0.69, *P* < 0.001; Spearman: rho = −0.52, *P* < 0.001; Fig. 2C) were both negatively correlated with the MMSE score. To exclude the potential effect of age on cognition, a linear regression model was used to predict the MMSE score based on the disruption indices adjusting for age (MMSE ∼ disruption indices + Age). The results show that only the disruption indices were significant (Age: *P* = 0.296, Global WM disruption: *P* < 0.001; Age: *P* = 0.425, Global brain region disruption: *P* < 0.001). The correlation test between age and MMSE score showed no significance (Pearson: r = −0.17, *P* = 0.156; Spearman: rho = −0.17, *P* = 0.176).

We further compared the patient subgroups stratified by the Karnofsky Performance Status (KPS) score of 80 as reported.^48^ We found that a worse KPS score was associated with higher disruptions of both brain regions and WM connections (both *P* < 0.001; Fig. 2B and D). We also found significant negative correlations between the KPS score and the global disruption indices (WM disruption: Pearson, *r* = −0.51, *P* < 0.001; Spearman: rho = −0.41, *P* < 0.001, Brain region disruption: Pearson, *r* = −0.49, *P* < 0.001; Spearman: rho = −0.31, *P* < 0.001). These results indicate that the stratified patient subgroups may have different global disruption indices.

Finally, we trained logistic regression models to predict the better or worse KPS as stratified previously. The baseline model including two preoperatively available variables: age and tumour volume, achieved an AUC of 0.79 [confidence interval (CI): 0.68–0.91]. Adding the global disruption indices into the model improved the AUC to 0.86 (CI: 0.77–0.94; Supplementary Fig. 1A). The out-of-sample prediction on the validation cohort showed improved performance (AUC = 0.71, CI: 0.53–0.89) compared to the baseline (AUC = 0.60, CI: 0.43–0.78; Supplementary Fig. 1B).

To rigorously assess the reproducibility of WM connection strength estimation, we compared the strengths of a cerebellar tract (middle cerebellar peduncle, MCP) that is not affected by the supratentorial tumours in our cohorts. No significant difference was found between controls and patients; Supplementary Fig. 2).

### Regional disrupted connectome characteristics

#### Tumour regions are more significantly disrupted than distant regions

The two study cohorts showed no significant difference in disruption indices (Supplementary Table 2). In comparing disruptions of WM connections, we observed significantly higher direct disruption (4.77 ± 1.56) than indirect disruption (2.59 ± 0.39, *P* < 0.001; Fig. 2E, F and Supplementary Table 3). Similarly for brain regions, focal tumour (CE: 5.83 ± 2.00; NE: 4.67 ± 1.33) were more significantly disrupted than the normal-appearing brain (distant: 2.90 ± 0.71, indirect: 2.56 ± 0.39, each *P* <0.001; Fig. 2G and H). The validation cohort showed similar disruption patterns. These results correspond to our understanding of tumour invasion and support the robustness of the regional disruption indices.

#### The regional disruptions are correlated with focal tumour volume

Pearson correlation tests showed that the WM connection disruptions in tumour (direct) and normal-appearing brain (indirect) were positively correlated (*r* = 0.44, *P* < 0.001). Similarly, the disruption of distant regions was positively correlated with that of tumour regions (distant versus CE: *r* = 0.43, *P* < 0.001; distant versus NE: *r* = 0.34, *P* = 0.028; Supplementary Table 4). Further, the tumour volume (measured by CE tumour) was positively correlated with the disruptions of both direct connections (*r* = 0.52, *P* < 0.001) and distant regions (*r* = 0.33, *P* < 0.001; Supplementary Table 5). Collectively, these data indicate that a larger focal tumour is associated with higher connectome disruption throughout the brain.

### The normal-appearing brain shows widespread disruption

We calculated the proportion of disrupted regions out of all brain regions. It is worth noting that, in the group analysis, a higher proportion of distant regions (16.8 ± 12.0%) was identified than focal lesion (CE: 5.8 ± 5.1%, *P* < 0.001), recapitulated by the validation cohort (Supplementary Table 6), indicating that the normal-appearing brain was widely disrupted.

At the group level, the average disruption probability of distant regions was higher (17.2 ± 9.0%) than focal lesion (CE: 11.8 ± 6.8%, *P* < 0.001), possibly due to the more extensive coverage of the distant regions. This finding further confirmed that the disruption of brain regions was widespread beyond the lesion.

**Figure 3:**
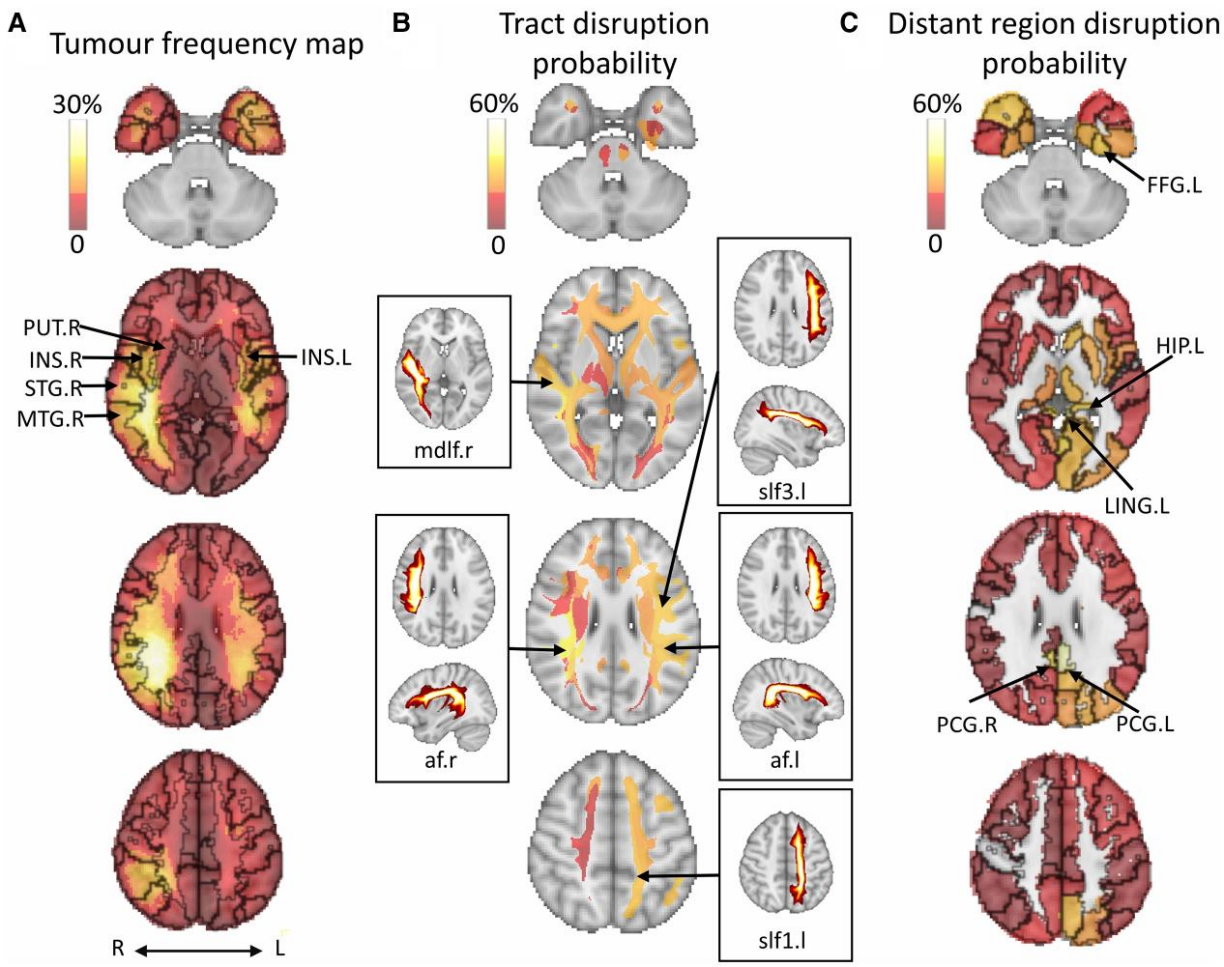
**Tumour density and disrupted anatomical structures.** (**A**) Tumour frequency maps are generated using tumour segmentation masks. The top five disrupted focal brain regions include the right superior temporal gyrus (STG.R), right middle temporal gyrus (MTG.R), right insula (INS.R), left insula (INS.L) and right lenticular nucleus, putamen (PUT.R). (**B**) The top five disrupted anatomical tracts and their maximum intensity projection: right arcuate fasciculus (af.r), right middle longitudinal fasciculus (mdlf.r), left superior longitudinal fasciculus 3 (slf3.l), left arcuate fasciculus (af.l) and left superior longitudinal fasciculus 1 (slf1.l). (**C**) The top five disrupted distant regions include the left posterior cingulate gyrus (PCG.L), right posterior cingulate gyrus (PCG.R), left lingual gyrus (LING.L), left fusiform gyrus (FFG.L) and left hippocampus. L/l = left; R/r = right. ***P < 0.001, **P < 0.01, *P < 0.05.

We further compared the tumour frequency map (Fig. 3A) with the distribution probability map of brain regions and tracts. Notably, the top five tracts most likely disrupted were mainly association tracts and close to the high-frequency regions (Fig. 3B and Supplementary Table 7), suggesting that the association tracts may be more vulnerable in glioblastoma patients. The top five most probably disrupted distant regions (Fig. 3C) were mainly in the low-frequency regions (see Supplementary Table 8 for details).

### Topological features and their association with distant regions

#### The focal tumour alters the topological property of the connectome

**Figure 4:**
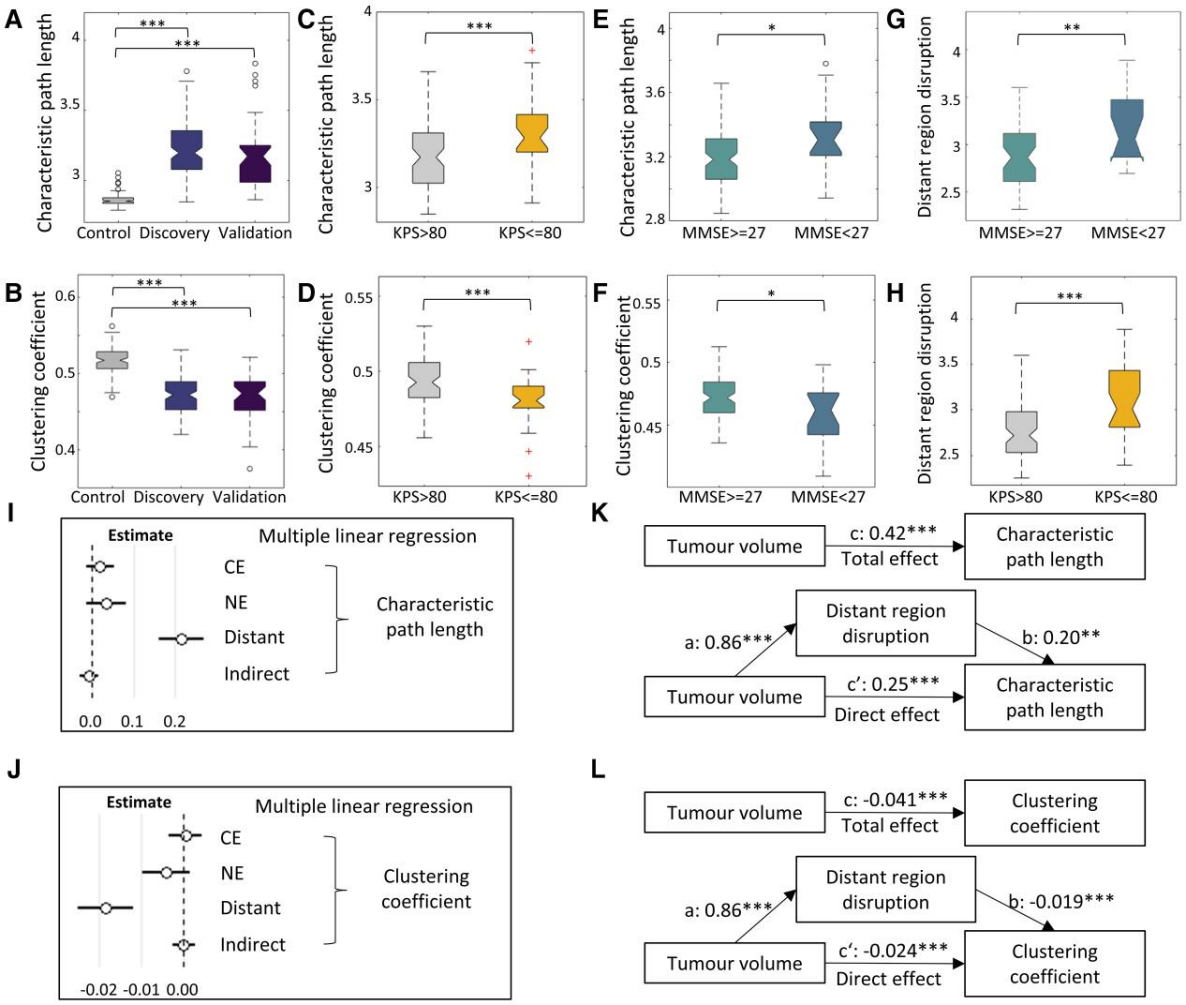
**Topological alteration of the connectome.** Patients show increased characteristic path length (**A**) and decreased clustering coefficient (**B**) compared to the controls. Patient subgroups with worse preoperative KPS (**C** and **D**) and MMSE (**E** and **F**) scores show increased characteristic path length and decreased clustering coefficient. Disruption of distant regions is higher in the subgroups with worse MMSE (**G**) and KPS (**H**), and it is the only significant predictor of characteristic path length (**I**) and clustering coefficient (**J**) in multiple linear regression. (**K**) The effects of tumour volume on characteristic path length are mediated by the disruption of distant regions: total effect (c path) = 0.42, *P* < 0.001; direct effect (c′ path) = 0.25, *P* < 0.001; mediation effect (c—c′) = 0.17, *P* = 0.008. (**L**) The effects of tumour volume on clustering coefficient are mediated by the disruption of distant regions: total effect (c path) = −0.041, *P* < 0.001; direct effect (c′ path) = −0.024, *P* < 0.001; mediation effect (c—c′) = −0.017, *P* < 0.001. ***P < 0.001, **P < 0.01, *P < 0.05.

We observed significantly higher characteristic path lengths in patient networks than healthy controls (*P* < 0.001; Fig. 4A). In contrast, the clustering coefficient of patients was significantly lower than that of healthy controls (*P* < 0.001; Fig. 4B and Supplementary Table 9). These results reveal that tumour lesions could dramatically alter the topology property of the structural connectome. No difference was found between topological features between two patient cohorts, which suggests the robustness of our approach.

We next determined the clinical significance of connectome topology by comparing the topological properties of the patient subgroups stratified by MMSE and KPS scores. We found that the patients with lower MMSE or KPS scores presented lower clustering coefficient (MMSE: *P* = 0.012, KPS: *P* < 0.001; Fig. 4C and E) and higher characteristic path length (MMSE: *P* = 0.013, KPS: *P* < 0.001; Fig. 4D and F). Moreover, characteristic path length (*r* = 0.43, *P* < 0.001) was positively correlated with tumour volume, while clustering coefficient (*r* = −0.45, *P* < 0.001) was negatively correlated with tumour volume, indicating that a larger focal lesion may have a greater influence on the connectome topology (Supplementary Table 10).

#### The disruption of distant regions is associated with the topological alteration

To understand the relation between the regional disruption with topological properties, we performed a multiple linear regression, which revealed that the disruption of distant regions was the only significant predictor of characteristic path length (estimate = 0.21, *P* < 0.001) and clustering coefficient (estimate = −0.018, *P* < 0.001; Fig. 4I and J).

We further performed the mediation analysis, which showed that tumour volume had both significant direct and indirect effects (mediated by the disruption of distant regions) on characteristic path length (direct: *P* < 0.001, indirect *P* = 0.008) and clustering coefficient (direct and indirect *P* < 0.001) (Fig. 4K and L). The findings were confirmed by the validation cohort (Supplementary Fig. 3).

We additionally compared the disruptions of distant regions in the subgroups stratified by the MMSE and KPS scores. We noticed that the patients with higher MMSE or higher KPS scores displayed significantly lower disruption of distant regions (Fig. 4G and H), consistent with the distinct network topological properties in these subgroups. The results further indicate the association between the disrupted distant regions and connectome topology.

### Topological features and disruption of distant regions are prognostic

For both disruption indices and topological features, we evaluated the prognostic value using log-rank tests and Cox proportional hazards models.

#### Log-rank test

**Figure 5:**
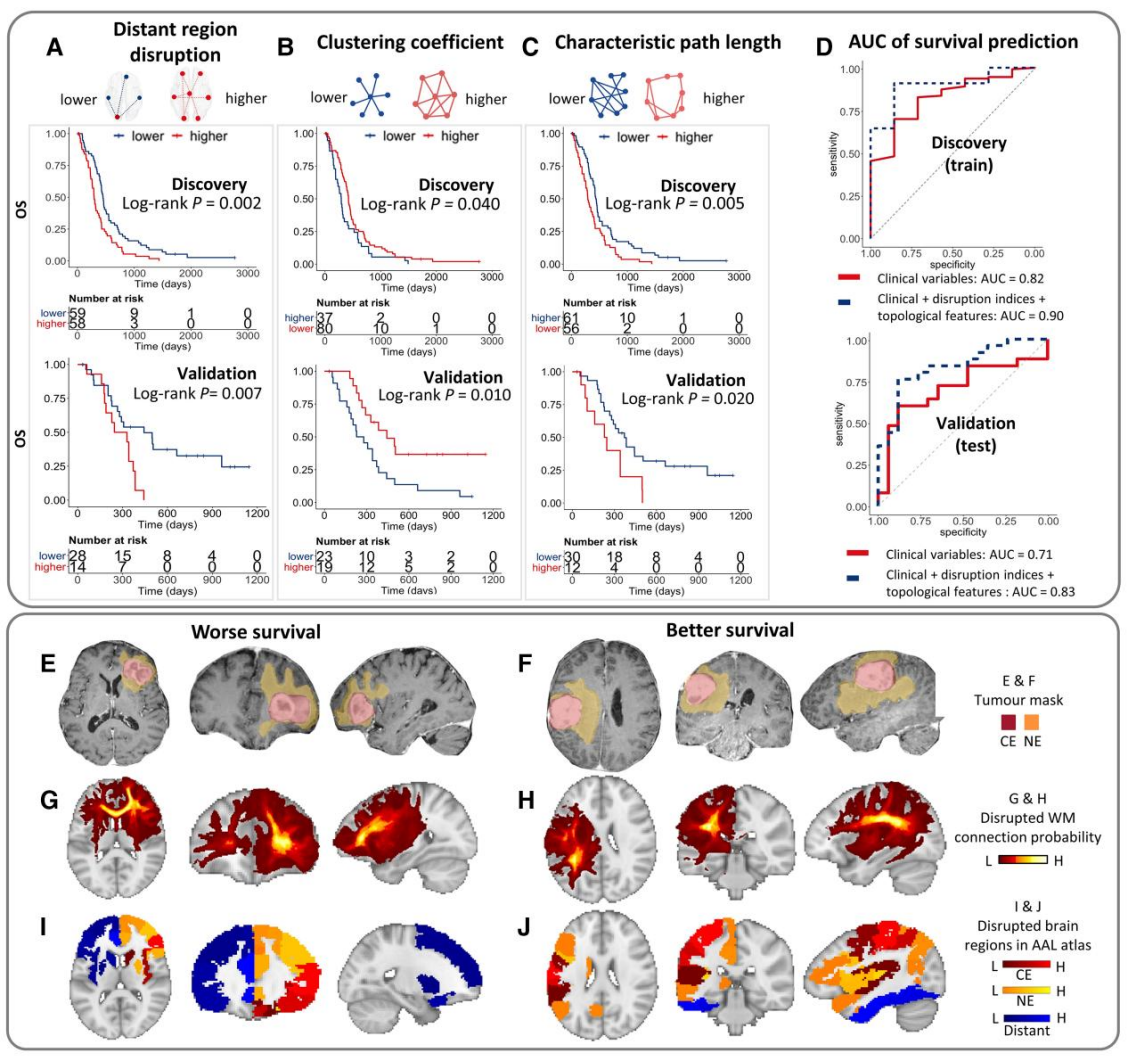
**The prognostic value of disruption indices and topological features.** The *top* shows that in both cohorts, higher disruption of distant regions (**A**), lower clustering coefficient (**B**) and higher characteristic path length (**C**) are associated with worse OS. (**D**) The model of predicting OS using clinical factors, disruption indices and topological features show improved AUC compared to clinical factors alone. *Bottom* shows two examples with worse or better survival (OS: 317 versus 1555 days; PFS: 159 versus 747 days). Both are IDH wild-type and MGMT unmethylated tumours of similar visible size in two males (aged 67 versus 69 years), who underwent complete resection followed by temozolomide chemoradiotherapy (**E** and **F**). Patients have similar tumour size on T_1C_ (23.6 versus 25.0 cm^3^). The patient with worse survival (**G**) has more widespread connection disruption beyond the visible lesion, compared to the patient with better survival (**H**). The disruption indices of distant regions (blue) are 3.0 (**I**) and 2.8 (**J**), respectively. Their topological features are distinct (clustering coefficient 0.44 versus 0.48: characteristic path length 3.31 versus 3.17). HR = hazard ratio.

In the subgroups stratified by the mean disruption of distant regions (2.9), patients with higher disruption had worse survival than lower disruption (OS: median 293 versus 449 days, *P* = 0.002, PFS: median 238 versus 307 days, *P* = 0.019; Fig. 5A). Further, the subgroups stratified by the optimal cut-off of topological features (clustering coefficient 0.46; characteristic path length 3.20) also had distinct survival. Precisely, the subgroup with a higher clustering coefficient had better survival than that with a lower clustering coefficient (OS: median 475 versus 294 days, *P* = 0.040, PFS: median 306 versus 238 days, *P* = 0.002; Fig. 5B). The subgroup with lower characteristic path length showed better survival than that with higher characteristic path length (OS: median 465 versus 288 days, *P* = 0.005, PFS: median 312 versus 244 days, *P* = 0.012; Fig. 5C). We further confirmed the findings in the validation cohort using identical cut-offs.

#### Cox proportional hazards modelling

From the Cox proportional hazards models (Table 1), we observed that higher disruptions of indirect connection (OS: HR = 1.36, *P* = 0.007; PFS: HR = 2.43, *P* = 0.046) and distant regions (OS: HR = 1.46, *P* = 0.049; PFS: HR = 1.49, *P* = 0.019) were associated with worse survival. For topological features, a higher clustering coefficient was associated with better survival (OS: HR = 0.63, *P* = 0.035; PFS: HR = 0.49, *P* = 0.002), while a higher characteristic path length was associated with worse survival (OS: HR = 1.56, *P* = 0.035; PFS: HR = 1.82, *P* = 0.009).

**Table 1:** Univariate survival statistics of the discovery cohort

	OS			PFS		
	HR	95%CI	*P*	HR	95%CI	*P*
**Clinical variables**						
Age	1.03	1.01 –1.05	**0**.**004**	1.03	1.00–1.05	**0**.**021**
Sex^a^	0.81	0.52–1.25	0.333	0.75	0.47–1.19	0.217
Performance^b^	1.60	1.09–2.36	**0**.**018**	1.63	1.04–2.55	**0**.**033**
IDH^c^	0.59	0.25–1.36	0.211	0.52	0.22–1.21	0.131
MGMT^d^	0.77	0.52–1.14	0.196	0.68	0.43–1.07	0.094
EOR^e^	1.89	1.26–2.84	**0**.**002**	1.90	1.19–3.03	**0**.**007**
Adjuvant treatment^f^	0.21	0.13–0.34	**<0**.**001**	0.22	0.11–0.41	**<0**.**001**
Tumour volume	1.01	1.00–1.01	**0**.**001**	1.01	1.00–1.02	**0**.**045**
Eloquent location^g^	0.93	0.64–1.36	0.711	1.06	0.69–1.62	0.807
Deep WM^h^	0.85	0.58–1.24	0.386	0.86	0.57–1.31	0.487
NE volume	0.87	0.72–1.05	0.135	0.87	0.70–1.07	0.192
**Disruption indices**						
Direct connection	1.07	0.87–1.30	0.790	1.08	0.88–1.32	0.464
Indirect connection	1.36	1.13–1.65	**0**.**007**	2.43	1.02–5.81	**0**.**046**
CE regions	0.95	0.86–1.04	0.667	0.96	0.86–1.06	0.418
NE regions	1.01	0.88–1.15	0.939	1.00	0.87–1.15	0.979
Distant regions	1.46	1.08–1.99	**0**.**049**	1.49	1.07–2.07	**0**.**019**
Indirect regions	1.06	0.87–1.29	0.790	0.80	0.83–1.28	0.795
**Topological features**						
Clustering coefficient	0.63	0.42–0.93	**0**.**035**	0.49	0.30–0.78	**0**.**002**
Characteristic path length	1.56	1.06–2.29	**0**.**035**	1.82	1.16–2.84	**0**.**009**

EOR = extent of resection. Bold values = *P* < 0.05.

Female as the reference.

KPS 90–100 as the reference.

IDH wild-type as reference.

Unmethylated MGMT as reference.

Incomplete resection as reference.

Concurrent chemoradiotherapy as reference.

Non-eloquent location as reference.

Affected deep WM as reference.

**Figure 6:**
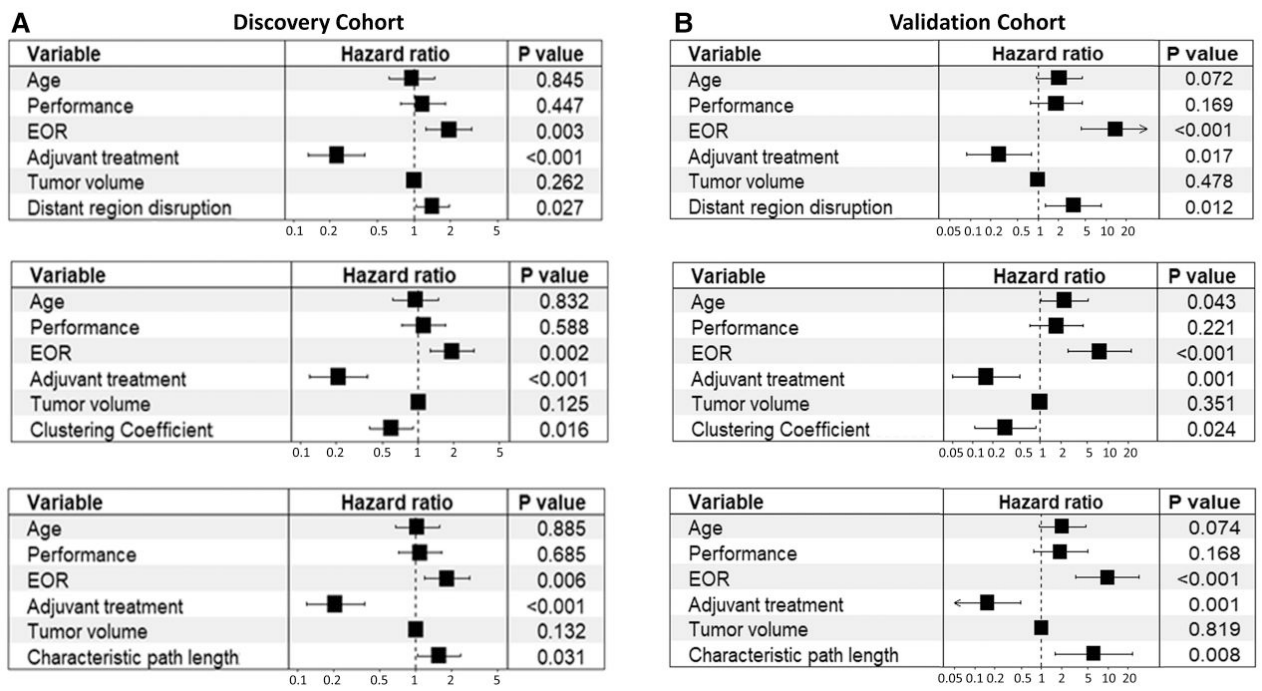
**Forest plots of multivariate survival modelling.** For the discovery (**A**) and validation (**B**) cohorts, the higher disruption of distant regions, higher characteristic path length and lower clustering coefficient are associated with worse survival. The prognostic value is independent of the significant clinical variables. EOR = extent of resection.

In the multivariate model adjusting for all the significant clinical covariates from the univariate models, the disruption of distant regions and topological features remained significant (Fig. 6 and Supplementary Table 11). Their prognostic value was confirmed by the validation cohort (Supplementary Tables 12 and 13).

#### Logistic model for survival prediction

We trained a logistic regression model on the discovery cohort to predict the 2-year OS using the disruption indices and topological features significant in the univariate Cox model. The baseline model, including the previous significant clinical variables (i.e. age, extent of resection and adjuvant therapy), achieved an AUC of 0.82 (CI 0.68–0.96). By adding the disruption indices and topological features into the baseline model, the AUC was improved to 0.90 (CI 0.80–0.99; Fig. 5D). We further tested this trained model in the validation cohort for out-of-sample validation, which confirmed the improved accuracy (AUC = 0.83, CI 0.71–0.96) by including disruption indices and topological features over the baseline model (AUC = 0.71, CI 0.54–0.87).

We presented two examples (Fig. 5E–J) with similar clinical variables but different disruption of distant regions, topological features and finally distinct survival (above and below the median, respectively).

### Preserved connectivity and recurrence distance associated with distant regions

#### The preserved connectome of distant regions indicates patient survival

**Figure 7:**
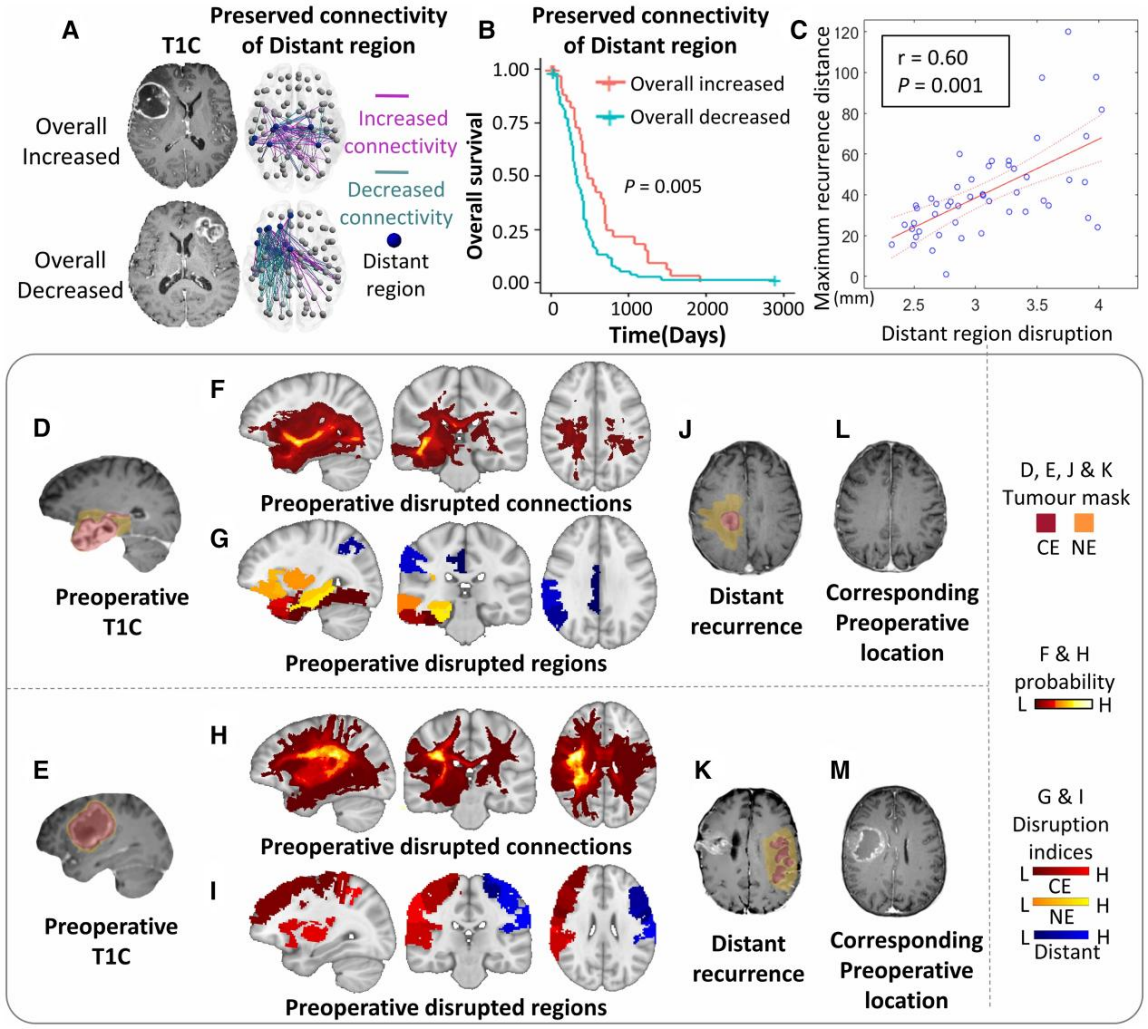
**The disruptions of the distant regions indicate survival significance and distant recurrence.** The top shows that after removing the disrupted WM connections of the distant regions, the preserved connections are categorized as increased or decreased connectivity in comparisons with healthy controls and aggregated to stratify patients. Two examples of overall increased and decreased connectivity are in **A**. The subgroup with overall increased connectivity shows better survival than overall decreased connectivity (**B**). The disruption index of the distant region is positively correlated with maximum recurrence distance (**C**). The bottom shows two examples of distant recurrence. Both patients present solitary visible lesions on preoperative T_1C_ images (**D** and **E**), widespread disrupted WM connections (**F** and **H**) and brain regions (**G** and **I**). In both patients, the distant recurrence location, either ipsilesional recurrence (**J**) or contralesional recurrence (**K**), corresponds to the distant regions (blue), which are linked to the primary lesion via the WM connections shown in **F** and **H**. A retrospective review of the preoperative T_1C_ images reveals no visible lesion in the recurrence location (**L** and **M**). T_1C_ = T_1_ contrast.

As the distant region disruption is the most significant biomarker, we further analysed its potential effect on the preserved connectome. First, we observed that 93.2% (109/117) patients displayed overall changes in connectivity. Among them, 24.7% (29/117) patients displayed overall increased connectivity, while 68.4% (80/117) patients showed overall decreased connectivity. We present two case examples with overall increased and decreased connectivity in the preserved connectome of the distant regions, respectively (Fig. 7A). The log-rank test showed that those patients with overall increased connectivity were associated with better survival (*P* = 0.005; Fig. 7B), confirmed by the validation cohort (Supplementary Fig. 4). The findings suggest that more integrated brain connectivity is associated with better patient survival, suggesting *a priori* more resilient connectome or network reorganization.

#### Disrupted connectome indicates tumour recurrence

Finally, we found that the higher distant region disruption was positively correlated with the furthest recurrence distance from tumour centroid (*r* = 0.60, *P* < 0.001; Fig. 7C). We present two cases that showed distant recurrence in follow-up scans, where the disrupted distant regions indicated occult tumour invasion invisible on the preoperative MRI (Fig. 7D–M).

## Discussion

The present study employed a connectome approach to investigate the disruption of structural connectivity in glioblastoma. Our main findings include: (i) glioblastomas cause widespread disrupted neural connectivity beyond the focal lesion; (ii) the disruption of the normal-appearing brain could mediate the alteration of connectome topology, associated with worse patient performance and affect patient survival; and (iii) the preserved connectome demonstrates evidence of network reorganization associated with survival.

The finding that glioblastomas can cause widespread structural impairment is in line with the previous studies using resting-state fMRI, reporting that glioma induced widespread functional impairment.^3,4^ The evidence supports that glioblastoma should be treated as a systematic disease rather than a local disease. Moreover, we found that only the disruption of distant regions was associated with topological alteration and patient survival among all the regional disruptions, suggesting the importance of characterizing global neural connectivity.

In the anatomical mapping of the disrupted connectome, we found that the top disrupted distant regions, e.g. posterior cingulate cortex and hippocampus, are essential structures of the limbic system, suggesting the propensity of the occult invasion affecting the limbic system. Moreover, the top affected anatomical tracts, e.g. arcuate fasciculus and superior longitudinal fasciculus, are long association tracts widely connecting separated gyri, suggesting that tumour invasion might spread through these tracts. Although at the macroscopic scale, our imaging findings may provide a perspective for previously reported neural–cancer interaction.^49^

We found that the connectivity measures could provide superior biomarkers for brain tumour stratification over conventional clinical factors, e.g. tumour location and volume. The network efficiency of the human brain generally reflects the integrity of brain function.^50^ Glioblastoma patients displayed decreased network efficiency compared to healthy controls, probably due to tumour disturbance on brain function. Interestingly, our results show that the preserved connectome demonstrates evidence of reorganization. The increased connectivity, indicating a more integrated network and more robust function, is associated with favourable survival. Although the mechanism remains further elucidated, it could suggest the opportunities of understanding neural–cancer interaction for patient prognosis.

Our study has important clinical implications. Due to the remarkable heterogeneity of glioblastoma, the development of quantitative prognostic markers is crucial for precise diagnosis and treatment. The structural connectome and topological features confer a novel approach to investigate the systematic changes of neural connectivity in glioblastoma. It could enable us to understand the interaction between tumour invasion and neural connectivity, which promises to stratify patients more precisely, help to develop targeted therapeutics and reduce neurological deficits. From a translational perspective, our approach shows the potential to quickly produce metrics to enhance prognosis determination and patient stratification through automated imaging analytical software in the clinic, incorporating various data processing and modelling procedures, which could assist clinical decision making in future. Additionally, tumour invasion in the normal-appearing brain visualized on the imaging could be integrated into the neuro-navigation and radiotherapy planning systems, offering more precise guidance for treatment targets.

Our study has limitations. First, structural connectome can only directly measure the connectivity of connecting tracts. Although most brain regions are connected via tracts, some functionally related regions may not be structurally connected. Future work could include resting-state MRI and functional connectivity. Second, we only included *de novo* glioblastoma who received first-line treatment in the trial. Molecular markers, i.e. IDH and MGMT methylation, were not significant as previously reported in our cohorts. Finally, neuronal degeneration, such as Wallerian Degeneration, may also lead to the disruptions of WM connections, which may not be differentiated by our approach. Nonetheless, the significant prognostic value of disruption indices could support the capability of our approach in indicating tumour invasion.

In conclusion, glioblastoma causes widespread impairment to the structural connectome. The invisible disruption on conventional MRI and connectome integrity are correlated with patient survival. Studying neural connectivity may provide a valuable tool for patient stratification and precise treatment for patients with brain tumours.

## Supplementary Material

awac360_Supplementary_Data

## References

[1] Ricard D , Idbaih A, Ducray F, Lahutte M, Hoang-Xuan K, Delattre J-Y. Primary brain tumours in adults. Lancet. 2012;379:1984–1996. 22510398 10.1016/S0140-6736(11)61346-9

[2] Cuddapah VA , Robel S, Watkins S, Sontheimer H. A neurocentric perspective on glioma invasion. Nat Rev Neurosci. 2014;15:455–465. 24946761 10.1038/nrn3765 PMC5304245

[3] Daniel AG , Park KY, Roland JL, et al Functional connectivity within glioblastoma impacts overall survival. Neuro Oncol. 2020;23:412–421. 10.1093/neuonc/noaa189 PMC7992880 32789494

[4] Stoecklein VM , Stoecklein S, Galiè F, et al Resting-state fMRI detects alterations in whole brain connectivity related to tumor biology in glioma patients. Neuro Oncol. 2020;22:1388–1398 32107555 10.1093/neuonc/noaa044 PMC7523460

[5] Aerts H , Fias W, Caeyenberghs K, Marinazzo D. Brain networks under attack: Robustness properties and the impact of lesions. Brain. 2016;139:3063–3083. 27497487 10.1093/brain/aww194

[6] Yu K , Lin CJ, Hatcher A, et al PIK3CA Variants selectively initiate brain hyperactivity during gliomagenesis. Nature. 2020;578:166–171. 31996845 10.1038/s41586-020-1952-2 PMC7577741

[7] Venkataramani V , Tanev DI, Strahle C, et al Glutamatergic synaptic input to glioma cells drives brain tumour progression. Nature. 2019;573:532–538. 31534219 10.1038/s41586-019-1564-x

[8] Price S , Burnet N, Donovan T, et al Diffusion tensor imaging of brain tumours at 3 T: A potential tool for assessing white matter tract invasion? Clin Radiol. 2003;58:455–462. 12788314 10.1016/s0009-9260(03)00115-6

[9] Price SJ , Pena A, Burnet NG, et al Tissue signature characterisation of diffusion tensor abnormalities in cerebral gliomas. Eur Radiol. 2004;14:1909–1917. 15221264 10.1007/s00330-004-2381-6

[10] Li C , Wang S, Yan JL, et al Characterizing tumor invasiveness of glioblastoma using multiparametric magnetic resonance imaging. J Neurosurg. 2019;132:1465–1472. 31026822 10.3171/2018.12.JNS182926

[11] Li C , Yan JL, Torheim T, et al Low perfusion compartments in glioblastoma quantified by advanced magnetic resonance imaging and correlated with patient survival. Radiother Oncol. 2019;134:17–24. 31005212 10.1016/j.radonc.2019.01.008 PMC6486398

[12] Li C , Wang S, Yan JL, et al Intratumoral heterogeneity of glioblastoma infiltration revealed by joint histogram analysis of diffusion tensor imaging. Neurosurgery. 2019;85:524–534. 30239840 10.1093/neuros/nyy388

[13] Yan JL , Li C, van der Hoorn A, Boonzaier NR, Matys T, Price SJ. A neural network approach to identify the peritumoral invasive areas in glioblastoma patients by using MR radiomics. Sci Rep. 2020;10:9748. 32546790 10.1038/s41598-020-66691-6 PMC7297800

[14] van Dijken BRJ , Yan JL, Boonzaier NR, et al Subventricular zone involvement characterized by diffusion tensor imaging in glioblastoma. World Neurosurg. 2017;105:697–701. 28642175 10.1016/j.wneu.2017.06.075

[15] Price SJ , Allinson K, Liu H, et al Less invasive phenotype found in isocitrate dehydrogenase-mutated glioblastomas than in isocitrate dehydrogenase wild-type glioblastomas: A diffusion-tensor imaging study. Radiology. 2017;283:215–221. 27849434 10.1148/radiol.2016152679

[16] Bullmore E , Sporns O. Complex brain networks: Graph theoretical analysis of structural and functional systems. Nat Rev Neurosci. 2009;10:186–198. 19190637 10.1038/nrn2575

[17] Rubinov M , Sporns O. Complex network measures of brain connectivity: Uses and interpretations. Neuroimage. 2010;52:1059–1069. 19819337 10.1016/j.neuroimage.2009.10.003

[18] Crossley NA , Mechelli A, Scott J, et al The hubs of the human connectome are generally implicated in the anatomy of brain disorders. Brain. 2014;137:2382–2395. 25057133 10.1093/brain/awu132 PMC4107735

[19] Raj A , LoCastro E, Kuceyeski A, et al Network diffusion model of progression predicts longitudinal patterns of atrophy and metabolism in Alzheimer’s disease. Cell Rep. 2015;10:359–369. 25600871 10.1016/j.celrep.2014.12.034 PMC5747552

[20] Sun Y , Chen Y, Collinson SL, Bezerianos A, Sim K. Reduced hemispheric asymmetry of brain anatomical networks is linked to schizophrenia: A connectome study. Cereb Cortex. 2017;27:602–615. 26503264 10.1093/cercor/bhv255

[21] Liu Y , Yang K, Hu X, et al Altered rich-club organization and regional topology are associated with cognitive decline in patients with frontal and temporal gliomas. Front Hum Neurosci. 2020;14:23. 32153374 10.3389/fnhum.2020.00023 PMC7047345

[22] Aerts H , Schirner M, Dhollander T, et al Modeling brain dynamics after tumor resection using The Virtual Brain. Neuroimage. 2020;213:116738. 32194282 10.1016/j.neuroimage.2020.116738

[23] Kinno R , Ohta S, Muragaki Y, Maruyama T, Sakai KL. Differential reorganization of three syntax-related networks induced by a left frontal glioma. Brain. 2014;137:1193–1212. 24519977 10.1093/brain/awu013

[24] Mandal AS , Romero-Garcia R, Hart MG, Suckling J. Genetic, cellular, and connectomic characterization of the brain regions commonly plagued by glioma. Brain. 2020;143:3294–3307. 33278823 10.1093/brain/awaa277 PMC7891236

[25] Liu L , Zhang H, Rekik I, Chen X, Wang Q, Shen D. Outcome prediction for patient with high-grade gliomas from brain functional and structural networks. Med Image Comput Comput Assist Interv. 2016;9901:26–34. 28649677 10.1007/978-3-319-46723-8_4 PMC5479332

[26] Gorlia T , van den Bent MJ, Hegi ME, et al Nomograms for predicting survival of patients with newly diagnosed glioblastoma: Prognostic factor analysis of EORTC and NCIC trial 26981-22981/CE. 3. Lancet Oncol. 2008;9:29–38. 18082451 10.1016/S1470-2045(07)70384-4

[27] Jenkinson M , Bannister P, Brady M, Smith S. Improved optimization for the robust and accurate linear registration and motion correction of brain images. Neuroimage. 2002;17:825–841. 12377157 10.1016/s1053-8119(02)91132-8

[28] Kamnitsas K , Ferrante E, Parisot S, et al DeepMedic for brain tumor segmentation. In: Crimi A and Bakas S, eds. Brainlesion: Glioma, Multiple Sclerosis, Stroke and Traumatic Brain Injuries: Springer; 2016:138–149.

[29] Schult T , Hauser T-K, Klose U, Hurth H, Ehricke H-H. Fiber visualization for preoperative glioma assessment: Tractography versus local connectivity mapping. PLoS ONE. 2019;14:e0226153. 10.1371/journal.pone.0226153 PMC6907809 31830068

[30] Smith SM , Jenkinson M, Johansen-Berg H, et al Tract-based spatial statistics: Voxelwise analysis of multi-subject diffusion data. Neuroimage. 2006;31:1487–1505. 16624579 10.1016/j.neuroimage.2006.02.024

[31] Fagerholm ED , Hellyer PJ, Scott G, Leech R, Sharp DJ. Disconnection of network hubs and cognitive impairment after traumatic brain injury. Brain. 2015;138:1696–1709. 25808370 10.1093/brain/awv075 PMC4614120

[32] Squarcina L , Bertoldo A, Ham TE, Heckemann R, Sharp DJ. A robust method for investigating thalamic white matter tracts after traumatic brain injury. Neuroimage. 2012;63:779–788. 22813952 10.1016/j.neuroimage.2012.07.016 PMC3471070

[33] Tzourio-Mazoyer N , Landeau B, Papathanassiou D, et al Automated anatomical labeling of activations in SPM using a macroscopic anatomical parcellation of the MNI MRI single-subject brain. Neuroimage. 2002;15:273–289. 11771995 10.1006/nimg.2001.0978

[34] Grabner G , Janke AL, Budge MM, Smith D, Pruessner J, Collins DL. Symmetric atlasing and model based segmentation: An application to the hippocampus in older adults. Med Image Comput Comput Assist Interv. 2006;9(Pt 2):58–66. 10.1007/11866763_8 17354756

[35] Avants BB , Tustison N, Song G. Advanced normalization tools (ANTS). Insight J. 2009;2:1–35.

[36] Behrens TE , Berg HJ, Jbabdi S, Rushworth MF, Woolrich MW. Probabilistic diffusion tractography with multiple fibre orientations: What can we gain? Neuroimage. 2007;34:144–155. 17070705 10.1016/j.neuroimage.2006.09.018 PMC7116582

[37] Andersson JL , Sotiropoulos SN. An integrated approach to correction for off-resonance effects and subject movement in diffusion MR imaging. Neuroimage. 2016;125:1063–1078. 26481672 10.1016/j.neuroimage.2015.10.019 PMC4692656

[38] Prasanna P , Mitra J, Beig N, et al Mass Effect Deformation Heterogeneity (MEDH) on gadolinium-contrast T1-weighted MRI is associated with decreased survival in patients with right cerebral hemisphere glioblastoma: A feasibility study. Sci Rep. 2019;9:1145. 30718547 10.1038/s41598-018-37615-2 PMC6362117

[39] Andersson JL , Jenkinson M, Smith S. Non-linear registration aka Spatial normalisation FMRIB Technical Report TR07JA2. FMRIB Analysis Group of the University of Oxford. 2007:1–22.

[40] Schwarz CG , Reid RI, Gunter JL, et al Improved DTI registration allows voxel-based analysis that outperforms tract-based spatial statistics. Neuroimage. 2014;94:65–78. 24650605 10.1016/j.neuroimage.2014.03.026 PMC4137565

[41] Ou Y , Akbari H, Bilello M, Da X, Davatzikos C. Comparative evaluation of registration algorithms in different brain databases with varying difficulty: results and insights. IEEE Trans Med Imaging. 2014;33:2039–2065. 24951685 10.1109/TMI.2014.2330355 PMC4371548

[42] Nyúl LG , Udupa JK, Zhang X. New variants of a method of MRI scale standardization. IEEE Trans Medical Imaging. 2000;19:143–150. 10784285 10.1109/42.836373

[43] Wei Y , Li C, Price SJ. Quantifying structural connectivity in brain tumor patients. In: Shen D, Liu T, Peters TM, et al, eds. Medical Image Computing and Computer Assisted Intervention – MICCAI 2019. Springer; 2021:519–529.

[44] Jenkinson M , Beckmann CF, Behrens T, Woolrich MW, Smith SM. FSL. Neuroimage. 2012;62:782–790. 21979382 10.1016/j.neuroimage.2011.09.015

[45] Watts DJ , Strogatz SH. Collective dynamics of ‘small-world’ networks. Nature. 1998;393:440–442. 9623998 10.1038/30918

[46] Roberts JA , Perry A, Roberts G, Mitchell PB, Breakspear M. Consistency-based thresholding of the human connectome. Neuroimage. 2017;145:118–129. 27666386 10.1016/j.neuroimage.2016.09.053

[47] Lin H , Zelterman D. Modeling survival data: extending the cox model. Taylor & Francis; 2002.

[48] Chaichana K , Parker S, Olivi A, Quiñones-Hinojosa A. A proposed classification system that projects outcomes based on preoperative variables for adult patients with glioblastoma multiforme. J Neurosurg. 2010;112:997–1004. 19817542 10.3171/2009.9.JNS09805

[49] Monje M , Borniger JC, D’Silva NJ, et al Roadmap for the emerging field of cancer neuroscience. Cell. 2020; 181:219–222. 32302564 10.1016/j.cell.2020.03.034 PMC7286095

[50] Sporns O , Zwi JD. The small world of the cerebral cortex. Neuroinformatics. 2004;2:145–162. 15319512 10.1385/NI:2:2:145

