# Supplementary material for: Structural connectome quantifies tumour invasion and predicts survival in glioblastoma patients
Source: Brain. 2022 Oct 3;146(4):1714–27. doi: 10.1093/brain/awac360 (PMC10115235; doi:10.1093/brain/awac360)
Supplement: awac360_Supplementary_Data [file awac360_supplementary_data.pdf]

Supplementary Materials and Methods

1. Patient inclusion and exclusion criteria

Inclusion Criteria: radiological diagnosis of *de novo* glioblastoma; Age 18 - 75; likely to be suitable for radiotherapy with concomitant and adjuvant temozolomide.

Exclusion Criteria: unsuitable for a contrast-enhanced MRI; poor image quality; pregnancy or plan for pregnancy; previous history of cranial surgery or cranial radiotherapy/chemotherapy; allergic to aminolaevulinic acid or suffering from porphyria; post-operative non-glioblastoma histology.

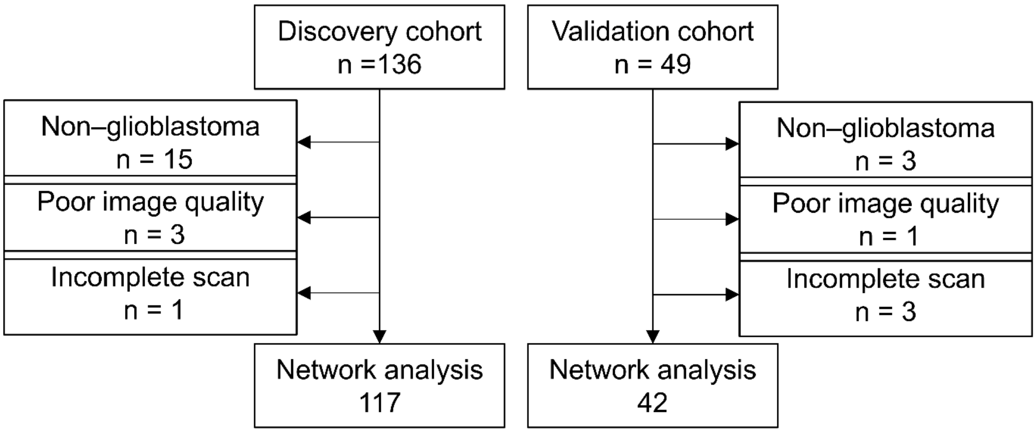

2. Scanning protocols of the patient cohorts

	**Discovery**	**Validation**
**T1-weighted imaging**		
Scanner	Siemens	GE
Sequence	MPRAGE	BRAVO
Field Strength (Tesla)	3	3
Flip Angle (degree)	9	12
Resolution X, Y, Z (mm)	1.0 × 1.0 × 1.0	1.0 × 1.0 × 1.0
FOV X × Y (mm)	256 × 240	256 × 256
Slice gap (mm)	0	0
TR (ms)	2300	8.16
TE (ms)	2.98	3.18
Inversion time (ms)	900	450
**T2-weighted imaging**		
Flip Angle (degree)	150	111
Resolution X, Y, Z (mm)	0.69 × 0.69 × 5	0.23 × 0.23 × 6
FOV X, Y (mm)	220 × 165	240 × 240
Slice gap (mm)	0.5	1
TR (ms)	4840	4942
TE (ms)	114	87.6
**T2-weighted fluid-attenuated inversion recovery (FLAIR)**		
Flip Angle (degree)	150	111
Resolution X, Y, Z (mm)	0.78 × 0.78 × 4.0	0.47 × 0.47 × 6.0
FOV X, Y (mm)	250 × 200	240 × 240
Slice gap (mm)	1	0
TR (ms)	7840	8000
TE (ms)	95	124.6
Inversion time (ms)	2500	2129
**Diffusion-weighted imaging**		
Flip Angle (degree)	90	90
Resolution X, Y, Z (mm)	2.0 × 2.0 × 2.0	0.93 × 0.93 × 3.0
FOV X, Y (mm)	192 × 192	256 × 128
Slice gap (mm)	0	3
TR (ms)	8300	8000
TE (ms)	98	87.4
B value	350, 650, 1000, 1300, 1600	1000
Gradient direction	12 for each b value	64

3. Demographics and imaging details of the ADNI healthy controls

The healthy controls used to generate the template of white matter connections were obtained from the Alzheimer's Disease Neuroimaging Initiative (ADNI) database (adni.loni.usc.edu). Ten elderly healthy subjects were selected from the ADNI-CN (healthy control) research group (mean age 60.9 ± 1.5 years, median age 60.2 years, range 58.4 - 62.1 years, three males). No significant age difference was found between the control and Discovery cohorts (Two-sample t-test $P = 0.712$). The diffusion MRI protocol of the healthy controls is as below.

Scanner	SIEMENS
Field Strength (Tesla)	3
Flip Angle (degree)	90
Resolution X, Y, Z (mm)	2,2,2
FOV X, Y, Z(mm)	115, 115, 80
Pulse Sequence	EP
TR (ms)	7200
TE (ms)	56
B value	1000
Gradient direction	54

4. Demographics and imaging details of IXI healthy subject dataset

The age-matched healthy controls used for generating control brain networks were obtained from the IXI dataset (https://brain-development.org/ixi-dataset/). The IXI dataset includes T1, T2, PD, dMRI, and MRA data for some or all of 619 healthy subjects.

For the present study, 117 age-matched healthy controls (mean age 59.9 ± 10.1years, median age 62.6 years, range 20.9-76.0 years, 63 males) were selected from the IXI dataset. Therefore, each patient in the Discovery cohort was matched with a corresponding healthy control with an age difference < 2 years. No significant age difference was found between the Discovery and healthy control cohorts (two-sample t-test, $P = 0.605$). The dMRI scans of the healthy controls were acquired in two hospitals using two Scanners:

	**Hammersmith Hospital**	**Guy's Hospital**
Scanner	Philips Medical Systems Intera	Philips Medical Systems Gyroscan Intera
Field Strength (Tesla)	3	1.5
Flip Angle (degree)	90	90
Resolution X, Y, Z (mm)	1.75, 1.75, 2.00	1.75, 1.75, 2.35
FOV X, Y (mm)	112, 110	112, 110
TR (ms)	11894.4	9054
TE (ms)	51	80
B value	1000	1000
Gradient direction	15	15

5. Descriptions of the topological features

Feature	Formula	Mathematical meaning	Interpretation
Clustering coefficient (Cl)	$$Cl = \frac{1}{N}\sum_{i\in N}\frac{2t_i}{k_i(k_i-1)},$$ where Cl is the average clustering coefficient in the networks, ki is the degree/strength of node i, and ti is the number of closed triangles attached to i. N is the number of nodes (*35*).	The probability that two nodes directly connected to a third node separately are also linked to each other (*35*).	A measure of functional segregation or specialization.
Characteristic path length (Lp)	$$Lp = \frac{1}{N(N-1)}\sum_{j\neq i} l_{ij},$$ where L is the average shortest path length between nodes in the network, lij is the shortest path length from node j to node i (*35*).	the average shortest path length between all possible pairs of nodes in a network (*35*).	A measure of global efficiency and network integration.
In brain networks, the node refers to brain regions and the edge refers to white matter connections between brain regions			

6. Brain regions in the Automatic Anatomical Labelling (AAL) atlas

Index	Left hemisphere abbr.	Right hemisphere abbr.	Region
1-2	PreCG.L	PreCG.R	Precental gyrus
3-4	SFGdor.L	SFGdor.R	Superior frontal gyrus, dorsolateral
5-6	ORBsup.L	ORBsup.R	Superior frontal gyrus, orbital part
7-8	MFG.L	MFG.R	Middle frontal gyrus mfg.l
9-10	ORBmid.L	ORBmid.R	Middle frontal gyrus, orbital part
11-12	IFGoperc.L	IFGoperc.R	Inferior frontal gyrus, opercular part
13-14	IFGtriang.L	IFGtriang.R	Inferior frontal gyrus, triangular part
15-16	ORBinf.L	ORBinf.R	Inferior frontal gyrus, orbital part
17-18	ROL.L	ROL.R	Rolandic operculum
19-20	SMA.L	SMA.R	Supplementary motor area
21-22	OLF.L	OLF.R	Olfactory cortex
23-24	SFGmed.L	SFGmed.R	Superior frontal gyrus, medial
25-26	ORBsupmed.L	ORBsupmed.R	Superior frontal gyrus, medial orbital
27-28	REC.L	REC.R	Gyrus rectus
29-30	INS.L	INS.R	Insula
31-32	ACG.L	ACG.R	Anterior cingulate and paracingulate gyri
33-34	DCG.L	DCG.R	Median cingulate and paracingulate gyri
35-36	PCG.L	PCG.R	Posterior cingulate gyrus
37-38	HIP.L	HIP.R	Hippocampus
39-40	PHG.L	PHG.R	Parahippocampal gyrus
41-42	AMYG.L	AMYG.R	Amygdala
43-44	CAL.L	CAL.R	Calcarine fissure and surrounding cortex
45-46	CUN.L	CUN.R	Cuneus
47-48	LING.L	LING.R	Lingual gyrus
49-50	SOG.L	SOG.R	Superior occipital gyrus
51-52	MOG.L	MOG.R	Middle occipital gyrus
53-54	IOG.L	IOG.R	Inferior occipital gyrus
55-56	FFG.L	FFG.R	Fusiform gyrus
57-58	PoCG.L	PoCG.R	Postcentral gyrus
59-60	SPG.L	SPG.R	Superior parietal gyrus
61-62	IPL.L	IPL.R	Inferior parietal, but supramarginal and angular gyri
63-64	SMG.L	SMG.R	Supramarginal gyrus
65-66	ANG.L	ANG.R	Angular gyrus
67-68	PCUN.L	PCUN.R	Precuneus
69-70	PCL.L	PCL.R	Paracentral lobule
71-72	CAU.L	CAU.R	Caudate nucleus
73-74	PUT.L	PUT.R	Lenticular nucleus, putamen
75-76	PAL.L	PAL.R	Lenticular nucleus, pallidum

77-78	THA.L	THA.R	Thalamus
79-80	HES.L	HES.R	Heschl gyrus
81-82	STG.L	STG.R	Superior temporal gyrus
83-84	TPOsup.L	TPOsup.R	Temporal pole: superior temporal gyrus
85-86	MTG.L	MTG.R	Middle temporal gyrus
87-88	TPOmid.L	TPOmid.R	Temporal pole: middle temporal gyrus
89-90	ITG.L	ITG.R	Inferior temporal gyrus
	R: right; L: left		

7. Anatomical tracts in the XTRACT atlas

Abbreviation		Tract
**Association Fibres**		
af.l	af.r	Arcuate fasciculus
fa.l	fa.r	Frontal aslant
ilf.l	ilf.r	Inferior longitudinal fasciculus
ifo.l	ifo.r	Inferior fronto-occipital fasciculus
mdlf.l	mdlf.r	Middle longitudinal fasciculuc
slf1.l	slf1.r	Superior longitudinal fasciculus 1
slf2.l	slf2.r	Superior longitudinal fasciculus 2
slf3.l	slf3.r	Superior longitudinal fasciculus 3
uf.l	uf.r	Uncinate fasciculus
vof.l	vof.r	Vertical occipital fasciculus
**Commissural Fibres**		
ac		Anterior commissure
fma		Forceps major
fmi		Forceps minor
mcp		Middle cerebellar peduncle
**Limbic Fibres**		
cbd.l	cbd.r	Cingulum subsection: Dorsal
cbp.l	cbp.r	Cingulum subsection: Peri-genual
cbt.l	cbt.r	Cingulum subsection: Temporal
fx.l	fx.r	Fornix
**Projection Fibres**		
ar.l	ar.r	Acoustic radiation
atr.l	atr.r	Anterior thalamic radiation
cst.l	cst.r	Corticospinal tract
or.l	or.r	Optic radiation
str.l	str.r	Superior thalamic radiation

r: right; l: left

Supplementary Results

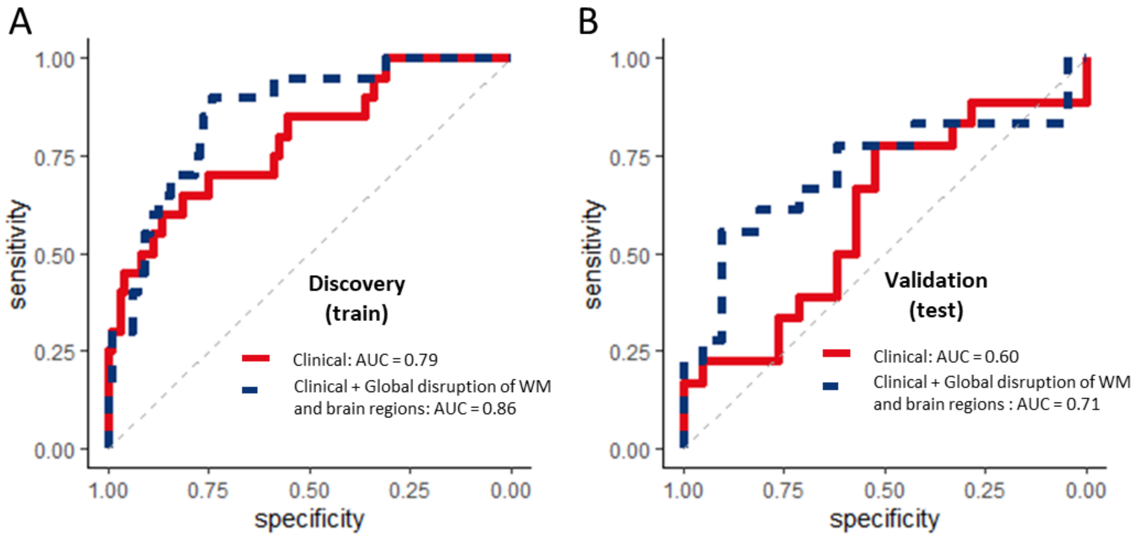

Fig. S1. Predicting KPS cut-off with a logistic model. (A) The logistic models trained on clinical data and clinical data (Age, tumor volume) with global disruption showed a slight increase in the AUC from 0.79 to 0.86 for the Discovery cohort. (**B**) The out-of-sample prediction on the Validation cohort showed a larger increase in the AUC from 0.60 to 0.71.

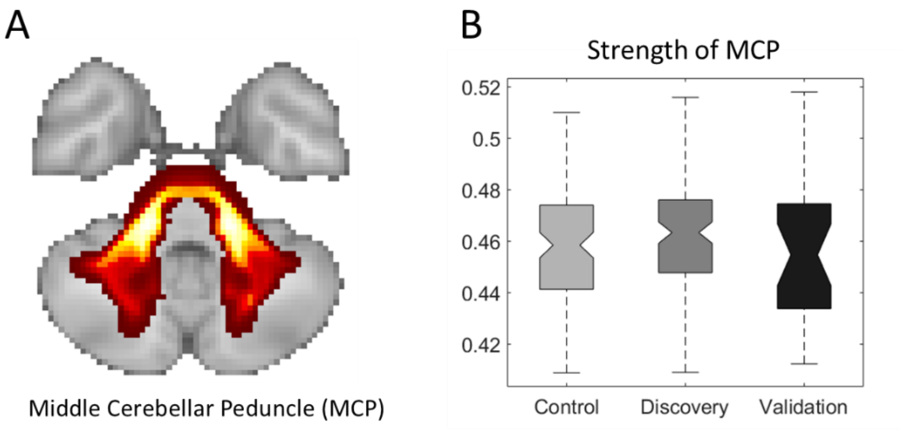

Fig. S2. White matter connection strengths of the middle cerebellar peduncle across three cohorts. **(A)** The middle cerebellar peduncle (MCP) is expected unaffected by the supratentorial tumour in the two study cohorts. **(B)** Patient and control cohorts show no significant difference in the MCP connection strength (Two-sample T test *P* value: Control vs Discovery = 0.063, Control vs Validation = 0.370; Two-sample F test *P* value: Control vs Discovery = 0.648, Control vs Validation = 0.757).

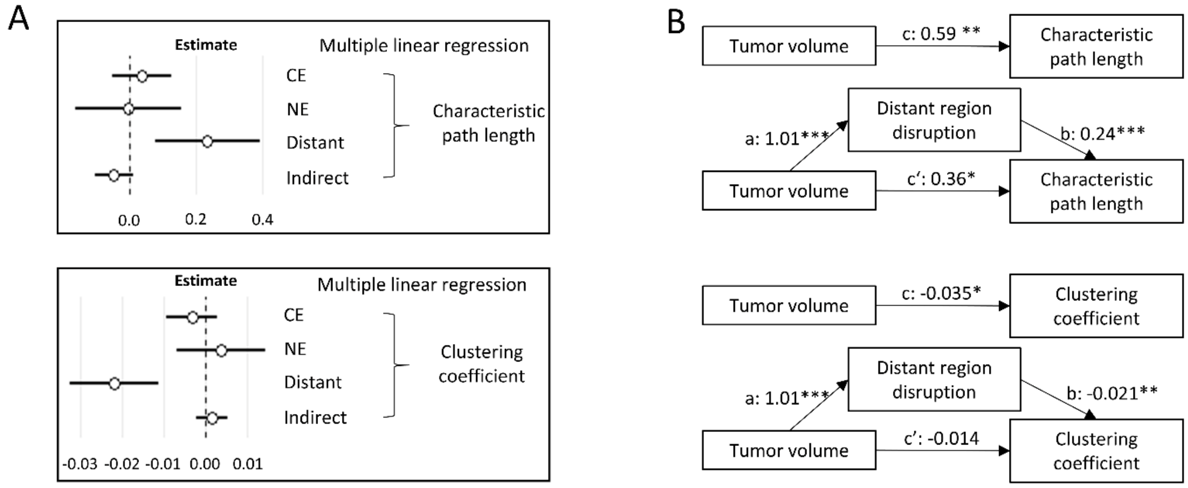

Fig. S3. Topological alteration of the structural connectome of the Validation cohort. (A) The disruption of the Distant regions is the only significant predictor for characteristic path length (estimate = 0.23, *P* = 0.005) **(upper)** and clustering coefficient (estimate = -0.022, *P* < 0.001) **(lower)** in multiple linear regression models. **(B)** The effects of tumour volume on characteristic path length are mediated by the disruption of the Distant regions **(upper)**: total effect (c path) = 0.59, *P* = 0.008; direct effect (c' path) = 0.36, *P* = 0.01; mediation effect (c – c') = 0.23, *P* < 0.001 (mediated by the disruption of the Distant regions). The effects of tumour volume on clustering coefficient are mediated by the disruption of the Distant regions **(lower)**: total effect (c path) = -0.035, *P* < 0.001; direct effect (c' path) = -0.014, *P* = 0.367; mediation effect (c – c') = -0.021, *P* =0.005 (mediated by the disruption of Distant regions). *P* values significant codes: *P* < 0.001 ***, *P* < 0.01 **, *P* < 0.05 *.

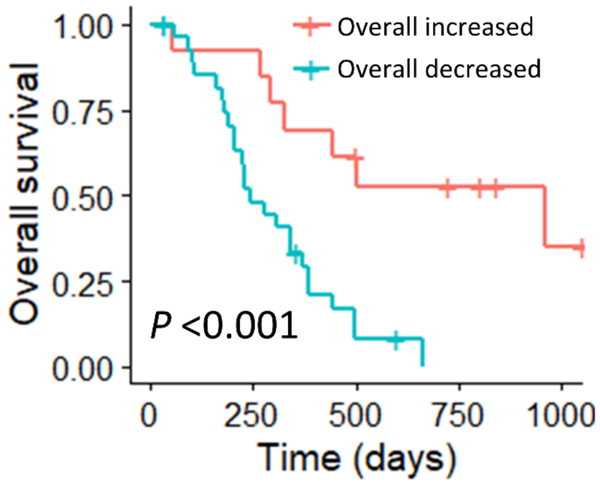

Fig. S4. Patient stratification in the Validation cohort using the preserved connectivity of Distant regions. Patients with overall increased strength (10/42, 23.8%) show better overall survival than the patients with decreased strength (32/42, 76.2%). Log-rank test: $P < 0.001$.

Table S1. Summary of the patient cohorts

Variable	Patient Number		*P* value
	**Discovery**	**Validation**	
**Age at diagnosis**			
<60	42	18	0.425
≥60	75	24	
**Sex**			
Male	89	30	0.236
Female	28	12	
**Extent of resection (of enhancing tumour)**			
Complete	77	32	0.572
Partial	32	6	
Biopsy	8	4	
**MGMT-methylation status [a]**			
Methylated	49	21	0.363
Unmethylated	68	21	
**IDH-1 mutation status**			
Mutant	7	1	0.360
Wild type	110	41	
**Pre-operative Tumour volumes(cm^3) [b]**			
Contrast-enhancing	44.7 ± 28.8	45.2 ± 29.4	0.841
Non-enhancing	50.3 ± 36.2	46.4 ± 32.6	0.652

(a): MGMT-methylation status unavailable for 4 patients in Discovery cohort; **(b)**: mean ± SD of original data. MGMT: O-6-methylguanine-DNA methyltransferase; IDH-1: Isocitrate dehydrogenase 1.

Table S2. Comparison of the two patient cohorts

	Discovery		Validation		
Category	Mean ± SD	95%CI	Mean ± SD	95%CI	*P* value
**White matter connection disruption**					
Direct	4.77 ± 1.56	2.60-7.43	4.95 ± 1.50	2.92-8.00	0.518
Indirect	2.59 ± 0.39	2.51-2.67	2.54 ± 0.53	2.43-2.65	0.520
**Brain region disruption**					
CE	5.83 ± 2.00	2.80-8.74	5.51 ± 1.80	2.28-7.45	0.363
NE	4.67 ± 1.33	2.77-7.43	4.54 ± 1.53	2.30-7.45	0.603
Distant	2.90 ± 0.71	2.30-3.83	3.08 ± 0.76	2.33-4.33	0.169
Indirect	2.56 ± 0.39	2.15-3.37	2.62 ± 0.55	2.17-4.05	0.447

Table S3. Comparison of regional disruption indices

White matter connection disruption				
	Discovery		Validation	
Category	Mean ± SD	*P* (vs Indirect)	Mean ± SD	*P* (vs Indirect)
Direct	4.77±1.56	<0.001	4.95±1.50	<0.001
Indirect	2.59±0.39	-	2.54±0.53	-

Brain region disruption								
	Discovery				Validation			
Category	Mean ± SD	*P* (vs NE)	*P* (vs Distant)	*P* (vs Indirect)	Mean ± SD	*P* (vs NE)	*P* (vs Distant)	*P* (vs Indirect)
CE	5.83±2.00	<0.001	<0.001	<0.001	5.51±1.80	0.018	<0.001	<0.001
NE	4.67±1.33	-	<0.001	<0.001	4.54±1.53	-	<0.001	<0.001
Distant	2.90±0.71	-	-	<0.001	3.08±0.76	-	-	0.018
Indirect	2.56±0.39	-	-	-	2.62±0.55	-	-	-

P value adjusted by FDR

Table S4. Pearson correlations of disruption indices

White matter connection disruption

	Discovery	Validation
Category	r (*P*)<br>Indirect	r (*P*)<br>Indirect
Direct	0.44 (<0.001)	0.51 (<0.001)

Brain region disruption

	Discovery			Validation		
Category	r<br>(*P*)<br>NE	r<br>(*P*)<br>Distant	r<br>(*P*)<br>Indirect	r<br>(*P*)<br>NE	r<br>(*P*)<br>Distant	r<br>(*P*)<br>Indirect
CE	0.41<br>(<0.001)	0.43<br>(<0.001)	0.21<br>(0.156)	0.63<br>(<0.001)	0.54<br>(0.002)	0.39<br>(0.094)
NE	-	0.34<br>(0.028)	0.26<br>(0.056)	-	0.54<br>(0.002)	0.42<br>(0.056)
Distant	-	-	0.11<br>(0.237)	-	-	0.22<br>(0.176)

P value adjusted by FDR

Table S5. Pearson correlations between connectome disruption and tumour volume

	Discovery		Validation	
	**Pearson r**	***P***	**Pearson r**	***P***
Direct connections	0.52	<0.001	0.72	<0.001
Indirect connections	0.07	0.453	0.09	0.563
CE regions	0.48	<0.001	0.62	<0.001
NE regions	0.20	0.041	0.52	0.002
Distant regions	0.33	<0.001	0.39	0.013
Indirect regions	-0.06	0.489	0.19	0.234

P value adjusted by FDR

Table S6. Proportions of the disrupted connectome

White matter connection disruptions

	Discovery		Validation	
Category	Mean ± SD	*P* (vs. Indirect)	Mean ± SD	*P* (vs. Indirect)
Direct	20.4 ± 9.6%	<0.001	20.7 ± 10.1%	<0.001
Indirect	11.5 ± 6.7%	-	13.2 ± 6.6%	-

Brain region disruptions

	Discovery				Validation			
Category	Mean ± SD	*P* (vs. NE)	*P* (vs. Distant)	*P* (vs. Indirect)	Mean ± SD	*P* (vs. NE)	*P* (vs. Distant)	*P* (vs. Indirect)
CE	5.8 ± 5.1%	<0.001	<0.001	<0.001	5.9 ± 4.0%	0.341	<0.001	<0.001
NE	11.3 ± 7.2%	-	<0.001	<0.001	8.6 ± 7.9%	-	0.007	<0.001
Distant	16.8 ± 12.0%	-	-	<0.001	16.3 ± 9.9%	-	-	<0.001
Indirect	3.2 ± 3.0%	-	-	-	2.1% ± 2.3%	-	-	-

P value adjusted by FDR

Table S7. Disruption probabilities of the XTRACT tracts

Index	Tracts	Disruption probability
1	ac	0.20
2	af.l	0.32
3	af.r	0.40
4	ar.l	0.23
5	ar.r	0.27
6	atr.l	0.27
7	atr.r	0.23
8	cbd.l	0.28
9	cbd.r	0.23
10	cbp.l	0.20
11	cbp.r	0.20
12	cbt.l	0.24
13	cbt.r	0.21
14	cst.l	0.25
15	cst.r	0.18
16	fa.l	0.29
17	fa.r	0.26
18	fma	-
19	fmi	0.27
20	fx.l	-
21	fx.r	-
22	ifo.l	0.28
23	ifo.r	0.30
24	ilf.l	-
25	ilf.r	-
26	mcp	-
27	mdlf.l	0.27
28	mdlf.r	0.38
29	or.l	0.23
30	or.r	0.29
31	slf1.l	0.30
32	slf1.r	-
33	slf2.l	0.30
34	slf2.r	-
35	slf3.l	0.32
36	slf3.r	-
37	str.l	0.22
38	str.r	0.17
39	uf.l	0.29
40	uf.r	0.29
41	vof.l	-

42	vof.r	-

P value adjusted by FDR. '-' denotes the tracts that are not significantly disrupted.

Table S8. Probabilities of CE, NE and Distant region disruption

	CE			NE			Distant		
Index	Mean ± SD	P	Prob.	Mean ± SD	P	Prob.	Mean ± SD	P	Prob.
1	0.49±0.38	0.001	0.11	0.49 ± 0.38	<0.001	0.14	0.46 ± 0.09	<0.001	0.15
2	0.53 ± 0.47	0.005	0.08	0.53 ± 0.47	0.001	0.10	0.59 ± 0.23	0.026	0.03
3	0.38 ± 0.23	0.003	0.08	0.38 ± 0.23	<0.001	0.14	0.43 ± 0.38	<0.001	0.18
4	0.66 ± 0.78	0.003	0.09	0.66 ± 0.78	0.001	0.11	0.51 ± 0.20	0.003	0.08
5	0.41 ± 0.24	0.017	0.05	0.41 ± 0.24	<0.001	0.15	0.23 ± 0.61	<0.001	0.13
6	0.74 ± 0.70	0.031	0.03	0.74 ± 0.70	0.011	0.05	0.42 ± 0.24	<0.001	0.14
7	0.81 ± 0.75	0.002	0.09	0.81 ± 0.75	<0.001	0.14	0.56 ± 0.48	<0.001	0.14
8	0.49 ± 0.44	<0.001	0.13	0.49 ± 0.44	0.001	0.10	0.53 ± 0.78	0.002	0.08
9	0.79 ± 1.11	0.014	0.05	0.79 ± 1.11	0.004	0.08	0.63 ± 0.17	0.006	0.06
10	0.28 ± 0.19	0.029	0.03	0.28 ± 0.19	0.004	0.07	0.35 ± 0.08	<0.001	0.21
11	0.18 ± 0.24	0.003	0.09	0.18 ± 0.24	<0.001	0.14	0.32 ± 0.63	<0.001	0.14
12	0.68 ± 0.41	0.001	0.10	0.68 ± 0.41	0.001	0.11	0.47 ± 0.21	0.005	0.08
13	0.32 ± 0.21	0.002	0.09	0.32 ± 0.21	<0.001	0.14	0.18 ± 0.04	<0.001	0.16
14	0.18 ± 0.17	0.001	0.10	0.18 ± 0.17	0.001	0.10	0.27 ± 0.73	<0.001	0.15
15	1.49 ± 1.64	0.009	0.06	1.49 ± 1.64	<0.001	0.19	0.80 ± 0.36	<0.001	0.16
16	0.51 ± 0.72	0.010	0.06	0.51 ± 0.72	0.001	0.12	0.30 ± 0.30	<0.001	0.24
17	0.47 ± 0.32	0.001	0.11	0.47 ± 0.32	<0.001	0.21	0.43 ± 0.11	<0.001	0.22
18	0.24 ± 0.21	<0.001	0.19	0.24 ± 0.21	<0.001	0.25	1.42 ± 1.24	0.005	0.06
19	0.64 ± 1.28	0.018	0.05	0.64 ± 1.28	0.005	0.07	1.00 ± 0.61	<0.001	0.12
20	0.39 ± 0.62	0.054	-	0.39 ± 0.62	0.021	0.04	0.79 ± 0.84	0.048	0.03
21	0.47 ± 0.47	0.031	0.03	0.47 ± 0.47	0.001	0.09	0.67 ± 1.03	<0.001	0.12
22	0.23 ± 1.05	0.007	0.06	0.23 ± 1.05	<0.001	0.15	0.90 ± 0.56	<0.001	0.22
23	0.39 ± 0.94	0.005	0.09	0.39 ± 0.94	0.001	0.12	0.68 ± 0.40	<0.001	0.16
24	0.24 ± 0.72	0.008	0.06	0.24 ± 0.72	0.001	0.10	1.06 ± 0.82	0.001	0.09
25	0.36 ± 0.86	0.024	0.04	0.36 ± 0.86	0.002	0.10	0.86 ± 0.49	0.003	0.07
26	0.51 ± 0.76	0.032	0.03	0.51 ± 0.76	0.004	0.08	0.68 ± 0.31	0.079	-
27	0.16 ± 0.55	0.029	0.03	0.16 ± 0.55	0.002	0.08	0.67 ± 0.81	0.001	0.09
28	0.68 ± 1.32	0.021	0.04	0.68 ± 1.32	0.006	0.06	0.98 ± 0.88	<0.001	0.23
29	0.50 ± 0.40	<0.001	0.21	0.50 ± 0.40	<0.001	0.35	0.17 ± 0.04	<0.001	0.24
30	0.53 ± 0.60	<0.001	0.30	0.53 ± 0.60	<0.001	0.43	0.69 ± 0.58	<0.001	0.12
31	0.59 ± 0.89	0.004	0.08	0.59 ± 0.89	<0.001	0.15	0.19 ± 0.13	0.002	0.09
32	0.49 ± 1.30	0.001	0.11	0.49 ± 1.30	<0.001	0.16	0.31 ± 0.15	0.001	0.10
33	0.51 ± 1.04	0.001	0.11	0.51 ± 1.04	<0.001	0.18	0.23 ± 0.18	<0.001	0.14
34	0.57 ± 0.87	0.003	0.08	0.57 ± 0.87	0.001	0.10	0.83 ± 0.41	0.014	0.04
35	0.13 ± 0.08	0.016	0.04	0.13 ± 0.08	0.006	0.06	0.15 ± 0.14	<0.001	0.46
36	0.51 ± 0.45	0.016	0.04	0.51 ± 0.45	0.001	0.09	0.92 ± 0.40	<0.001	0.35
37	0.79 ± 1.00	<0.001	0.14	0.79 ± 1.00	<0.001	0.21	0.93 ± 0.71	<0.001	0.32
38	0.02 ± 0.31	<0.001	0.23	0.02 ± 0.31	<0.001	0.29	0.61 ± 0.79	<0.001	0.21
39	0.05 ± 0.27	0.001	0.11	0.05 ± 0.27	<0.001	0.18	0.85 ± 0.62	<0.001	0.27

40	0.59 ± 1.84	<0.001	0.15	0.59 ± 1.84	<0.001	0.15	0.09 ± 0.36	<0.001	0.26
41	1.15 ± 2.12	0.002	0.09	1.15 ± 2.12	<0.001	0.14	0.28 ± 0.21	<0.001	0.22
42	1.29 ± 1.01	0.003	0.09	1.29 ± 1.01	<0.001	0.14	0.79 ± 1.14	<0.001	0.25
43	1.62 ± 1.74	0.016	0.04	1.62 ± 1.74	0.002	0.08	0.46 ± 0.33	<0.001	0.29
44	0.91 ± 0.84	<0.001	0.18	0.91 ± 0.84	<0.001	0.26	0.65 ± 1.18	<0.001	0.17
45	1.09 ± 1.25	0.002	0.09	1.09 ± 1.25	<0.001	0.11	0.89 ± 0.64	<0.001	0.23
46	1.57 ± 1.53	<0.001	0.18	1.57 ± 1.53	<0.001	0.22	1.15 ± 0.51	<0.001	0.13
47	1.97 ± 1.68	0.009	0.05	1.97 ± 1.68	0.001	0.10	1.37 ± 0.88	<0.001	0.34
48	1.86 ± 1.30	<0.001	0.12	1.86 ± 1.30	<0.001	0.16	1.17 ± 0.98	<0.001	0.21
49	2.26 ± 2.25	0.004	0.08	2.26 ± 2.25	<0.001	0.12	1.39 ± 0.72	<0.001	0.28
50	1.90 ± 1.31	<0.001	0.18	1.90 ± 1.31	<0.001	0.24	0.96 ± 1.22	<0.001	0.16
51	2.73 ± 2.24	0.001	0.11	2.73 ± 2.24	<0.001	0.17	1.12 ± 0.94	<0.001	0.25
52	2.79 ± 2.44	<0.001	0.15	2.79 ± 2.44	<0.001	0.21	1.33 ± 1.24	<0.001	0.11
53	2.11 ± 1.93	0.030	0.03	2.11 ± 1.93	0.001	0.09	1.30 ± 1.47	<0.001	0.24
54	2.46 ± 2.68	0.001	0.09	2.46 ± 2.68	<0.001	0.16	1.26 ± 0.68	<0.001	0.23
55	1.36 ± 1.26	<0.001	0.15	1.36 ± 1.26	<0.001	0.22	1.04 ± 0.48	<0.001	0.32
56	1.01 ± 0.99	<0.001	0.21	1.01 ± 0.99	<0.001	0.22	0.89 ± 1.24	<0.001	0.25
57	2.67 ± 1.76	0.004	0.08	2.67 ± 1.76	0.001	0.11	1.09 ± 0.72	0.008	0.06
58	1.69 ± 1.20	0.001	0.11	1.69 ± 1.20	<0.001	0.17	0.68 ± 0.31	0.079	-
59	1.00 ± 0.88	0.003	0.08	1.00 ± 0.88	0.001	0.09	0.92 ± 1.38	<0.001	0.24
60	3.11 ± 2.80	0.001	0.12	3.11 ± 2.80	<0.001	0.18	1.55 ± 1.02	<0.001	0.18
61	1.97 ± 2.31	0.001	0.09	1.97 ± 2.31	<0.001	0.12	1.07 ± 0.93	0.004	0.07
62	1.45 ± 1.19	<0.001	0.21	1.45 ± 1.19	<0.001	0.27	1.21 ± 0.55	0.008	0.05
63	1.16 ± 0.99	0.007	0.07	1.16 ± 0.99	<0.001	0.13	1.66 ± 1.79	0.001	0.10
64	1.69 ± 2.53	<0.001	0.21	1.69 ± 2.53	<0.001	0.29	1.51 ± 1.26	0.005	0.06
65	1.18 ± 1.60	0.001	0.10	1.18 ± 1.60	<0.001	0.13	1.38 ± 1.49	0.001	0.09
66	1.61 ± 1.69	<0.001	0.21	1.61 ± 1.69	<0.001	0.23	1.33 ± 2.04	0.006	0.06
67	1.10 ± 2.41	0.002	0.09	1.10 ± 2.41	<0.001	0.15	1.53 ± 1.34	<0.001	0.29
68	1.32 ± 2.16	<0.001	0.21	1.32 ± 2.16	<0.001	0.29	1.30 ± 1.07	<0.001	0.15
69	1.06 ± 2.05	0.052	-	1.06 ± 2.05	0.006	0.06	1.58 ± 1.74	0.006	0.06
70	1.35 ± 2.02	0.087	-	1.35 ± 2.02	0.006	0.06	1.43 ± 1.16	0.009	0.05
71	1.55 ± 1.92	0.001	0.10	1.55 ± 1.92	<0.001	0.16	1.22 ± 0.95	<0.001	0.30
72	0.88 ± 1.83	0.001	0.10	0.88 ± 1.83	<0.001	0.19	1.25 ± 1.61	<0.001	0.14
73	1.77 ± 2.68	<0.001	0.14	1.77 ± 2.68	<0.001	0.25	1.51 ± 1.70	<0.001	0.26
74	1.91 ± 1.32	<0.001	0.19	1.91 ± 1.32	<0.001	0.30	0.74 ± 0.29	<0.001	0.20
75	1.93 ± 1.80	0.160	-	1.93 ± 1.80	0.016	0.04	1.29 ± 1.28	<0.001	0.21
76	1.94 ± 1.98	0.004	0.08	1.94 ± 1.98	<0.001	0.14	0.74 ± 0.56	<0.001	0.23
77	2.04 ± 2.80	0.083	-	2.04 ± 2.80	0.001	0.09	0.98 ± 0.62	<0.001	0.29
78	1.58 ± 2.20	0.004	0.07	1.58 ± 2.20	<0.001	0.17	0.78 ± 0.74	<0.001	0.21
79	1.96 ± 1.88	0.001	0.11	1.96 ± 1.88	<0.001	0.21	1.34 ± 1.02	0.055	-
80	0.87 ± 0.67	<0.001	0.22	0.87 ± 0.67	<0.001	0.28	0.62 ± 0.63	0.001	0.10
81	1.64 ± 1.45	<0.001	0.21	1.64 ± 1.45	<0.001	0.28	1.53 ± 1.04	0.001	0.10
82	2.16 ± 2.26	<0.001	0.34	2.16 ± 2.26	<0.001	0.37	1.68 ± 1.50	0.002	0.08

83	0.21 ± 1.19	<0.001	0.14	0.21 ± 1.19	<0.001	0.24	1.24 ± 1.61	<0.001	0.20
84	0.35 ± 1.06	<0.001	0.16	0.35 ± 1.06	<0.001	0.21	1.39 ± 1.28	<0.001	0.26
85	1.78 ± 3.90	<0.001	0.22	1.78 ± 3.90	<0.001	0.29	0.57 ± 1.14	<0.001	0.15
86	2.53 ± 3.36	<0.001	0.31	2.53 ± 3.36	<0.001	0.34	0.90 ± 0.77	<0.001	0.12
87	3.61 ± 2.64	0.001	0.11	3.61 ± 2.64	<0.001	0.17	1.71 ± 2.11	<0.001	0.17
88	3.31 ± 2.84	0.001	0.11	3.31 ± 2.84	<0.001	0.14	1.19 ± 0.93	<0.001	0.32
89	2.86 ± 2.56	<0.001	0.17	2.86 ± 2.56	<0.001	0.23	1.54 ± 1.87	<0.001	0.25
90	2.66 ± 2.58	<0.001	0.22	2.66 ± 2.58	<0.001	0.28	1.78 ± 1.46	<0.001	0.19

P value adjusted by FDR. '-' denotes the brain regions that are not significantly disrupted across the group

Table S9. Topological features of healthy controls and patient cohorts

Topological features	Group	Mean ± SD	95% CI	*P* (vs. Validation)	*P* (vs. Control)
Clustering coefficient	Discovery	0.47 ± 0.03	0.43 - 0.52	1.000	<0.001
	Validation	0.47 ± 0.03	0.41 - 0.52	-	<0.001
	Control	0.52 ± 0.02	0.48 - 0.55	-	-
Characteristic path length	Discovery	3.22 ± 0.20	2.93 - 3.57	1.000	<0.001
	Validation	3.18 ± 0.24	2.88 - 3.73	-	<0.001
	Control	2.86 ± 0.04	2.80 - 2.93	-	-

P value adjusted by FDR

Table S10. Pearson correlations between topological features and tumour volume

	Discovery		Validation	
	**Pearson r**	***P***	**Pearson r**	***P***
Clustering coefficient	-0.45	<0.001	-0.49	0.002
Characteristic path length	0.43	<0.001	0.58	<0.001

P value adjusted by FDR

Table S11. Multivariate survival statistics of the Discovery cohort

Feature	OSN			PFSN		
	HR	95%CI	*P* value	HR	95%CI	*P* value
Age	1.03	0.66-1.59	0.907	1.18	0.73-1.91	0.507
Performance[a]	1.19	0.77-1.84	0.432	1.21	0.72-2.02	0.474
EOR[b]	1.68	1.07-2.64	0.024	1.97	1.19-3.27	0.008
Adjuvant treatment[c]	0.24	0.14-0.41	<0.001	0.27	0.13-0.55	<0.001
Tumour volume	1.01	1.00-1.02	0.102	1.00	0.99-1.01	0.352
**Indirect connections**	1.32	1.07-1.61	**0.009**	1.17	0.95-1.44	0.146
Age	0.96	0.62-1.48	0.845	0.99	0.61-1.61	0.976
Performance[a]	1.18	0.77-1.81	0.447	1.27	0.76-2.14	0.365
EOR[b]	1.96	1.26-3.03	0.003	2.25	1.35-3.74	0.002
Adjuvant treatment[c]	0.23	0.13-0.39	<0.001	0.23	0.11-0.47	<0.001
Tumour volume	1.01	1.00-1.02	0.262	1.00	0.99-1.01	0.940
**Distant regions**	1.43	1.04-1.95	**0.027**	1.58	1.11-2.24	**0.011**
Age	0.95	0.61-1.49	0.832	1.10	0.67-1.79	0.709
Performance[a]	1.13	0.73-1.73	0.588	1.14	0.69-1.88	0.620
EOR[b]	1.96	1.27-3.01	0.002	2.09	1.27-3.41	0.003
Adjuvant treatment[c]	0.21	0.12-0.37	<0.001	0.26	0.12-0.54	<0.001
Tumour volume	1.01	1.00-1.02	0.125	1.00	0.99-1.01	0.552
**Clustering coefficient**	0.59	0.38-0.91	**0.016**	0.53	0.32-0.88	**0.013**
Age	1.03	0.67-1.59	0.885	1.16	0.72-1.87	0.550
Performance[a]	1.09	0.71-1.68	0.685	1.08	0.65-1.80	0.766
EOR[b]	1.85	1.20-2.85	0.006	1.97	1.19-3.25	0.008
Adjuvant treatment[c]	0.21	0.12-0.37	<0.001	0.24	0.11-0.49	<0.001
Tumour volume	1.01	1.00-1.02	0.132	1.00	0.99-1.01	0.594
**Characteristic path length**	1.59	1.04-2.43	**0.031**	1.58	0.97-2.57	0.067

(a). KPS Performance > 70 as the reference; **(b)**. Complete resection as the reference; **(c)** Non-CCRT as the reference. *P* value adjusted. **EOR**: extent of resection, **KPS**: Karnofsky Performance Score; **CCRT**: temozolomide concurrent chemoradiotherapy. **Bold *P* values** are still significant after adjusted by FDR

Table S12. Univariate survival statistics of the Validation cohort

Feature	OS			PFS		
	HR	95%CI	*P* value	HR	95%CI	*P* value
**Clinical variables**						
Age	1.05	1.01-1.09	0.018	1.05	1.00-1.10	0.016
Sex[a]	0.99	0.47-2.09	0.973	1.55	0.68-3.50	0.294
Performance[b]	1.58	0.78-3.22	0.205	0.82	0.39-1.71	0.595
IDH[c]	0.63	0.09-4.67	0.652	0.36	0.05-2.75	0.327
MGMT[d]	0.61	0.30-1.24	0.178	0.44	0.20-0.95	**0.036**
EOR[e]	8.69	3.42-22.1	**<0.001**	3.35	1.06-10.5	**0.039**
Adjuvant treatment[f]	0.15	0.07-0.36	**<0.001**	0.28	0.11-0.73	**0.010**
Tumour volume	1.00	1.00-1.00	0.790	1.00	1.00-1.00	0.545
Eloquent location[g]	0.65	0.23-1.85	0.416	0.71	0.28-1.80	0.467
Deep white matter[h]	0.42	0.19-0.94	**0.036**	1.85	0.80-4.30	0.150
Non-enhancing volume	1.33	0.90-1.96	0.159	1.30	0.87-1.96	0.198
**Disruption indices**						
Direct connection	1.10	0.78-1.57	0.577	1.09	0.74-1.61	0.659
Indirect connection	1.22	0.44-3.41	0.702	3.62	1.25-10.5	**0.018**
CE region	1.02	0.83-1.26	0.833	1.01	0.78-1.30	0.954
NE region	1.01	0.82-1.25	0.896	0.99	0.77-1.28	0.969
Distant region	3.36	1.50-7.53	**0.003**	2.03	1.10-3.77	**0.025**
Indirect region	0.92	0.70-1.24	0.614	0.94	0.72-1.24	0.667
**Topological features**						
Clustering coefficient	0.43	0.21-0.88	**0.021**	0.28	0.10-0.75	**0.012**
Characteristic path length	3.61	1.21-10.8	**0.021**	5.75	1.49-22.2	**0.011**

(a). Female as the reference; **(b)**. KPS Performance > 70 as the reference; **(c)**. IDH wildtype as the reference; **(d)** Unmethylated MGMT as the reference; **(e)**. Complete resection as the reference; **(f)** Non-CCRT as the reference. **(g).** Non-eloquent location as reference. **(h)** Deep white matter tracts affected as reference. **EOR**: extent of resection. **KPS**: Karnofsky Performance Score; **CCRT**: temozolomide concurrent chemoradiotherapy. *P* value adjusted by FDR

Table S13. Multivariate survival statistics of the Validation cohort

Feature	OS			PFS		
	HR	95%CI	*P* value	HR	95%CI	*P* value
Age	1.81	0.82-4.03	0.143	2.62	1.12-6.13	0.026
Performance[a]	1.82	0.72-4.62	0.205	4.00	1.46-11.0	0.007
EOR[b]	10.0	3.21-31.4	<0.001	7.01	1.75-28.1	0.006
Adjuvant treatment[c]	0.15	0.05-0.44	0.001	0.16	0.04-0.61	0.007
Tumour volume	1.00	0.98-1.02	0.922	1.01	0.99-1.03	0.326
**Indirect connections**	1.30	0.90-1.87	0.163	2.20	1.39-3.47	**0.001**
Age	2.05	0.94-4.49	0.072	3.01	1.28-7.12	0.012
Performance[a]	1.86	0.77-4.52	0.169	3.26	1.21-8.83	0.020
EOR[b]	14.0	4.36-45.2	<0.001	5.31	1.39-20.4	0.015
Adjuvant treatment[c]	0.26	0.09-0.79	0.017	0.42	0.09-1.83	0.246
Tumour volume	0.99	0.98-1.01	0.478	1.00	0.98-1.02	0.934
**Distant regions**	3.36	1.30-8.67	**0.012**	2.47	1.08-5.66	**0.033**
Age	2.31	1.03-5.18	0.043	2.88	1.19-6.97	0.019
Performance[a]	1.75	0.71-4.32	0.221	2.41	0.94-6.13	0.066
EOR[b]	7.60	2.56-22.6	<0.001	2.24	0.47-10.7	0.311
Adjuvant treatment[c]	0.16	0.05-0.49	0.001	0.27	0.07-1.05	0.059
Tumour volume	0.99	0.98-1.01	0.351	1.00	0.98-1.02	0.797
**Clustering coefficient**	0.30	0.11-0.85	**0.024**	0.28	0.06-1.25	0.095
Age	2.08	0.93-4.63	0.074	2.50	1.07-5.88	0.035
Performance[a]	1.94	0.76-4.96	0.168	2.72	1.04-7.14	0.042
EOR[b]	9.88	3.30-29.6	<0.001	3.22	0.78-13.3	0.106
Adjuvant treatment[c]	0.16	0.05-0.49	0.001	0.21	0.05-0.87	0.031
Tumour volume	1.00	0.99-1.02	0.819	1.01	0.99-1.03	0.384
**Characteristic path length**	6.16	1.61-23.5	**0.008**	7.89	1.40-44.5	**0.019**

(a). KPS Performance = 0 as the reference; **(b)**. Complete resection as the reference; **(c)** Non-CCRT as the reference. *P* value adjusted. **EOR**: extent of resection, **KPS**: Karnofsky Performance Score; **CCRT**: temozolomide concurrent chemoradiotherapy. **Bold *P* values** are still significant after adjusted by FDR
